# Reactor-based approach for achieving high-silica zeolites: a fed-batch strategy

**DOI:** 10.1039/d5ta08521g

**Published:** 2026-04-27

**Authors:** Amirhossein Javdani, Gleb Ivanushkin, Ahmed Sajid, Iqtidar Ali Khan, Ibrahim Khalil, Paola Herrero, Quanli Ke, Aron Deneyer, Michiel Dusselier

**Affiliations:** a Center for Sustainable Catalysis and Engineering (CSCE), KU Leuven 3001 Heverlee Belgium michiel.dusselier@kuleuven.be; b Université Lyon 1, CNRS, IRCELYON, UMR 5256 Villeurbanne France

## Abstract

High-silica zeolites are challenging to synthesize in hydroxide media using conventional batch reactors. This study advances beyond traditional batch crystallization methods by exploring a reactor-based strategy for tailoring zeolite properties through mid-way synthesis intervention. By employing the Fed-batch (FB) reactor strategy, we demonstrate that controlled addition of silica precursors at critical synthesis stages, without interrupting temperature or pressure conditions, grants an alternative pathway to high-silica zeolites. Two model systems were investigated: the transformation of FAU-to-CHA and FAU-to-LEV *via* interzeolite conversion (IZC). These controlled manipulations resulted in an elevated Si/Al ratio and distinct structural properties (*e.g.*, internal silanols) in the final zeolites. The resulting materials were exhaustively characterized and tested in CO_2_ conversion, highlighting the potential of reactor-based approaches for designing tailored high-performance zeolite catalysts.

## Abstract 2

Distinct features of the Fed-batch reactor facilitate the synthesis of high silica zeolites, which are key elements in sustainable catalysis.

## Graphical abstract

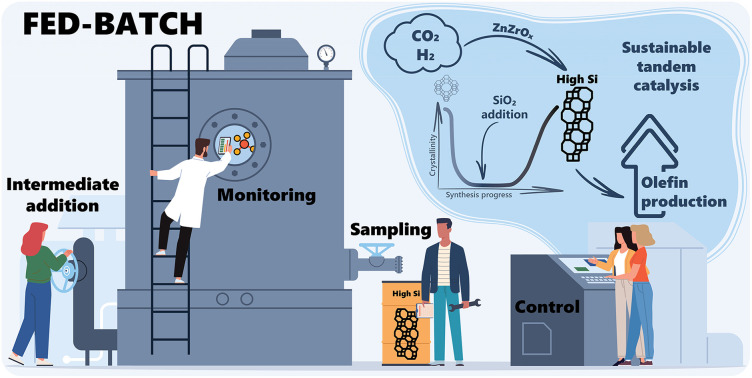

## 1. Introduction

Zeolites are (micro)porous materials with a crystalline structure, composed of TO_4_ (T = Si, Al) tetrahedral units. Inherently, zeolites exhibit high tunability, characterized by adjustable Si/Al ratios, acidity strengths, pore architectures, and crystal morphology. This imparts significant versatility and facilitates their integration into diverse industrial applications.^1,2^ Conventionally, zeolite synthesis is performed by mixing (most often amorphous) precursor materials, setting them into a batch-type autoclave, and applying heat under controlled conditions until the desired (metastable) crystalline phase has grown. Although significant advances have been made in various zeolite synthesis techniques, most progress has stemmed from ingredient-based approaches, primarily through *ab initio* variations in starting batch compositions.^3^ However, recent reports have highlighted the possibility of mid-way changes in batch composition. Robijns *et al.*, in our group, introduced bottom-up control over the Si/Al ratio of CHA and MFI zeolites through the “split synthesis” concept, which involves stopping the synthesis after a certain time, removing the Si-rich liquid or Al-rich solid phase, and allowing the remaining mixture to react further to achieve the final product.^4^ In another report by Shibuya *et al.*, the intermediate addition of an organic structure-directing agent (OSDA) has been used as a way to control its competitive effect to obtain CON zeolite.^5^ In their dual-OSDA strategy, introducing both OSDAs from the beginning led to competition and an undesired phase, whereas starting with one and adding the other at later stages shifted the competition into cooperation, resulting in a pure CON phase.^6^

While these examples are promising, their conventional “cooling-opening-heating” procedure causes side effects (*e.g.*, T-dependent precipitation) and could still be improved. The ability to change composition and manipulate zeolite formation under operational temperature and pressure without disrupting the synthesis is missing. Such disruptions might lead to uncontrolled supersaturation and undesired phase transformations, which are factors that significantly influence crystallization pathways and final material properties. Therefore, reactor-based solutions would enable us to overcome temporal effects (cooling-reheating) and implement an *in situ* strategy to impact concentration profiles along a synthesis. Moreover, time-dependent parameters could be more effectively tracked and monitored through improved reactor design directly during the synthesis.

There are recent reports on Electro-Assisted Synthesis (EAS) of heteroatom-containing zeolites *via in situ* electric anodic oxidation.^7–10^ In bottom-up batch approaches, the presence of metal precursors, such as Sn, Zn, and Ti, in initial zeolite mixtures can interfere with synthesis steps and hinder nucleation. Ivanushkin *et al.* overcame this challenge and achieved immense Lewis acid site densities (*via* framework metal incorporation) by designing novel reactors that enable precise timed-dosing of heteroatoms during synthesis *via* controlled electrochemical release. In a different reactor-based strategy, we developed a platform for monitoring and controlling zeolite synthesis, known as the “Fed-batch” (FB) reactor, and provided a proof-of-concept for controlled heteroatom release through an intermediate feeding.^11^ On top, this work has demonstrated the FB reactor capability in time-mapping of zeolite synthesis and identifying its key crystallization steps (*e.g.*, the onset of crystallization) through the sampling feature, as well as manipulating the synthesis through the feeding feature and the intermediate addition of heteroatom precursors at operational temperatures and pressures. In the current study, we extend this strategy toward achieving high-silica zeolites, using the FB configuration as a platform for mid-synthesis intervention.

High-silica zeolites are valued for their improved hydrothermal stability, increased hydrophobicity, and desirable catalytic properties.^12,13^ However, while several distinct methods are available in the literature,^14–17^ synthesizing zeolites with high Si/Al ratios in certain framework types remains challenging under conventional hydrothermal conditions, *i.e.*, hydroxide media. Such Si-rich zeolites are often achieved through fluoride-mediated synthesis routes, which involve safety concerns or require prolonged synthesis durations. Additionally, these methods may suffer from limited silicon incorporation efficiency, particularly in frameworks that are less favorable (or less known) for high-silica crystallization. For certain applications, such as the methanol to hydrocarbon conversion (coupled with CO_2_ hydrogenation to methanol or not), increasing the Si/Al ratio (*e.g.*, for CHA topology) proved to improve catalyst stability and lifetime.^18–20^ Increasing the Si/Al ratio of zeolites was achieved through bottom-up (direct synthesis) or top-down approaches (*e.g.*, dealumination). For instance, Jin *et al.* performed dealumination on CHA zeolite made without organics to get a high-silica CHA and achieved a longer lifetime and increased olefin selectivity.^21^

Interzeolite conversion (IZC) is a synthesis approach in which one zeolite (parent) is transformed into another (daughter) by rearranging its framework under specific conditions. Over the past decade, this method has gained momentum due to its ability to produce a wide range of industrially relevant zeolites from readily available precursors.^3,22^ The synthesis in IZC involves several stages, including dissolution (I), induction (II), crystallization (III), and maturation (IV).^23^ There is clearly a complicated breaking and reforming of T–O bonds involved.^24^ Notably, the evolution of some IZC systems (following I to IV) features a “transient window”, where both parent and daughter zeolite phases are absent, indicating an almost complete transformation into soluble oligomeric species (and sometimes a small fraction of amorphous solids) before the formation of the new crystalline structure.^25^ This mid-synthesis window, a region of uncertainty (in terms of knowing exactly what happens), could be used as a “point of intervention” in the FB strategy to manipulate the IZC outcome. The choice of addition of reagents in this transient window thus comes down to three factors: (1) High supersaturation at this point leads to a large thermodynamic drive for the formation of zeolite crystals, (2) the pH of the synthesis mixture reaches its lowest point during the transient window (evidenced in our prior work^4^), allowing for slowed dissolution of the added Si which speeds up as the crystallization of the zeolite releases OH, causing T-atoms to be selectively released from the added Si during growth (when needed most) and (3) the presence of some nuclei inside of the synthesis mixture can also selectively guide the synthesis towards a certain product. In many IZC syntheses, the Si/Al ratio of the resulting zeolite roughly that of the parent material or is much lower.^26,27^ Here, we demonstrate the capabilities of the FB platform to manipulate IZC and enhance the silica content of useful zeolites. Specifically, we provide an in-depth proof of this concept for the FAU-to-CHA synthesis, reaching Si/Al product ratios ranging from 64 to 103. We further proved the broader applicability of the method on the FAU-to-LEV transformation. All the FB-made zeolites were studied in detail using various characterization techniques, and the most promising ones were applied in the coupled CO_2_ hydrogenation-acid-catalyzed methanol conversion.

## 2. Materials and methods

### 2.1. Reactor design

**Fig. 1:**
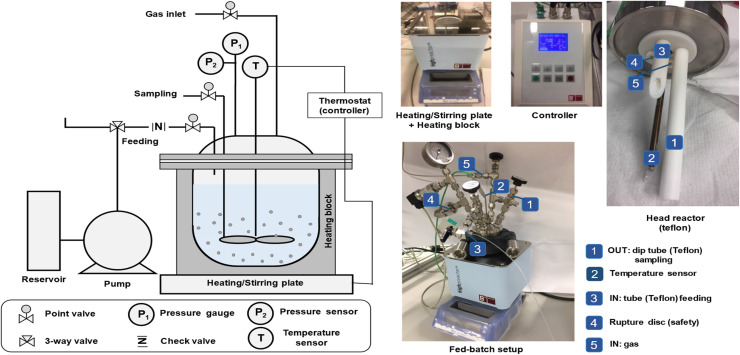
The technical scheme of FB reactor (left) and pictures of the interior and different parts of the set-up (right).

Zeolite synthesis was carried out in a custom-made FB set-up (100 ml) with a Teflon interior and feeding/sampling tubes (Fig. 1). The reactor (BR-100 reactor of Berghof) consists of a lid, made of SS316 Ti with a PTFE lining, and a vessel, also made of SS316 Ti, with the possibility of inserting a Teflon cup. Samples can be taken during synthesis through a liquid sampling PTFE dip tube (present in the synthesis medium). Besides, ingredients can also be added during synthesis *via* a PTFE tube, which is located above the synthesis medium. Limited blockage and stable low feeding rates (minimum 20 µl min^−1^) are key features of the peristaltic pump (SF-10 reagent pump by Vapourtec) for adding different types of solutions (such as suspensions or viscous solutions) while operating at temperature and under pressure (<10 bar). Considering feeding and sampling, the operating volume of the FB ranges from 10–60 ml. The lower limit of 10 ml ensures efficient agitation (magnetic stirring) and accurate measurement of the actual temperature. The upper limit of 60 ml ensures a safe working environment, allowing droplets of ingredients to be added always above the synthesis medium instead of in it. A unique feature of this setup is that temperature (*T*) and pressure (*P*) are constantly logged *via* sensors inside the synthesis medium, while these parameters are typically guesswork in a conventional batch zeolite synthesis in ovens (for *T* this is heavily the case in the startup phase due to thermal lag). Here, profiles can be immediately plotted and logged on a monitor (BTC-3000 temperature controller and data logger software of Berghof). It is essential to adjust the reactor's PID (proportional–integral–derivative) parameters according to the operating temperature to prevent sudden temperature fluctuations. In this work, PID parameters were set to 5-125-200 for operation at 160 °C. Practical rules for sampling, feeding, and maintenance were reported in our previous work.^11^

### 2.2. Experimental procedures

#### 2.2.1. FAU-to-CHA interzeolite conversion

**Scheme 1:**
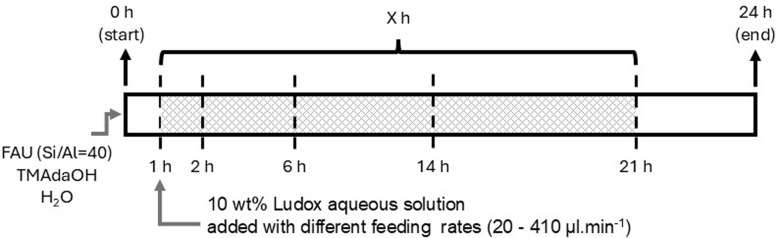
Schematic illustration of the timed-addition procedure in synthesis of CHA zeolite with FB reactor. Feeding duration or *X* could be 1 (so to 2 h), 5 (to 6 h), 13 (to 14 h), or 20 h (to 21 h).

Typically, pure CHA zeolite phase (with high Si/Al ratio) was prepared by mixing USY CBV780 (Si/Al = 40, or SiO_2_/Al_2_O_3_ = 80), an aqueous solution of 20 wt% trimethyladamantylammonium hydroxide (TMAdaOH), and MilliQ water. This mixture was loaded into a 100 ml Berghof Teflon liner and stirred for 16 h at room temperature (outside of the metal body). After this aging step, the liner was loaded into the reactor, closed and placed into the heating block. Interzeolite conversion/crystallization was conducted at 160 °C for 24 hours (same as most IZC systems for CHA synthesis) under constant stirring (900 rpm), while the intermediate addition was performed after 1 h from starting the synthesis, a solution of LUDOX colloidal silica (10 wt%) was pumped with different feeding rates (20–410 µl min^−1^) into the reactor, while being at the desired temperature (160 °C) and under autogenous pressure. The colloidal silica solutions were freshly prepared on each FB run and were constantly pumped for *X* hours (where *X* is 1, 5, 13, or 20) in total (Scheme 1). Finally, the reactor was immediately quenched in an ice-water bath. Once the system reached ambient temperature, the product mixture was collected. Then, the solid part was recovered by centrifugation (9500 rpm, ≥3 min), washed several times with MilliQ water (18.2 MΩ) until the pH value of the decanted water reached 9, and finally washed with acetone, then dried overnight at 100 °C. If required, dry samples were calcined to remove the organic template in a muffle furnace under airflow at 580 °C for about 6 h with a ramping time of 9 h (1 °C min^−1^ heating rate).

For comparison, one sample was synthesized in the FB reactor with the *ab initio* addition of colloidal silica to the synthesis mixture (*ab initio* addition), and another sample was synthesized without intermediate feeding (IZC without feeding) but still in the FB, similar to the classic batch system (batch mode).

The following ratios illustrate the chemical batch composition changes, which are determined by the feeding function (normalized by 1 mol of silica):

• Starting composition: 1 SiO_2_: 0.025 AlO_2_^−^: 0.35 TMAdaOH: 25 H_2_O (starting volume: 25 ml, theoretical Si/Al: 40).

• Composition after feeding: 1 SiO_2_: 0.012 AlO_2_^−^: 0.17 TMAdaOH: 27.47 H_2_O (final volume: 50 ml, theoretical Si/Al: 83).

#### 2.2.2. FAU-to-LEV interzeolite conversion

**Scheme 2:**
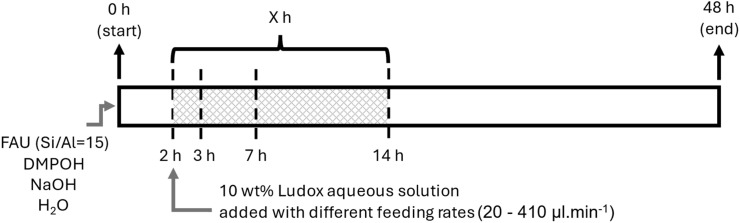
Schematic illustration of the timed-addition procedure in synthesis of LEV zeolite with FB reactor. Feeding duration or *X* could be 1 (so to 3 h), 5 (to 7 h), or 12 h (to 14 h).

Pure LEV zeolite phase (with an elevated Si/Al ratio) was prepared by mixing USY CBV720 (Si/Al = 15, or SiO_2_/Al_2_O_3_ = 30), sodium hydroxide, an aqueous solution of 20 wt% *N*,*N*-dimethylpiperidinium hydroxide (DMPOH), and MilliQ water. DMPOH was prepared by ion-exchange treatment using *N*,*N*-dimethylpiperidinium chloride and an ion-exchange resin, Amberlite™ IRN78. This mixture was loaded into a 100 ml Berghof Teflon liner and stirred for 16 h at room temperature (outside the metal body). After this aging step, the liner was loaded into the reactor, closed, and placed into the heating block. Interzeolite conversion/crystallization was conducted at 160 °C for 48 hours (same as most IZC systems using DMPOH for LEV synthesis) under constant stirring (900 rpm), while the intermediate addition was performed 2 h after starting the synthesis, a solution of LUDOX colloidal silica (10 wt%) was pumped with different feeding rates (20–250 µl min^−1^) into the reactor, while being at the desired temperature (160 °C) and under autogenous pressure. The colloidal silica solutions were freshly prepared for each FB run and were constantly pumped for *X* hours (where *X* is 1, 5, or 12) in total (Scheme 2). Finally, the reactor was immediately quenched in an ice-water bath. After crystallization, the product was treated as detailed in Subsection 2.2.1.

For comparison, one sample was synthesized in the FB reactor with the *ab initio* addition of colloidal silica to the synthesis mixture (*ab initio* addition), and another sample was synthesized without intermediate feeding (IZC without feeding), similar to the classic batch system (batch mode).

The following ratios illustrate the chemical batch composition changes, which are determined by the feeding function (normalized by 1 mol of silica):

• Starting composition: 1 SiO_2_: 0.066 AlO_2_^−^: 0.2 DMPOH: 0.3 NaOH: 10 H_2_O (starting volume: 15 ml, theoretical Si/Al: 15).

• Composition after feeding: 1 SiO_2_: 0.045 AlO_2_^−^: 0.13 DMPOH: 0.2 NaOH: 16.6 H_2_O (final volume: 30 ml, theoretical Si/Al: 22).

#### 2.2.3. Time-mapping of zeolite synthesis

All the above-mentioned cases were also synthesized with just the starting composition (no feeding) in the FB reactor, tracked, and time-mapped using the sampling feature. A detailed sampling procedure is described in our previous paper.^11^ In short, some essential parameters should be taken into account, such as ensuring homogeneity of mixture, avoiding temperature/pressure drop, and taking a proportional sample volume with respect to the whole synthesis medium. Samples (3 ml sample size) were taken during synthesis without cooling the reactor and treated (washed and dried) as described in Section 2.2.1. PXRD analysis was performed on the dry samples to track the zeolite formation.

#### 2.2.4. Acid treatment of zeolite

Post-synthesis acid treatment was applied to CHA zeolites, to investigate the role of unstable silica species. The treatment was conducted using a 0.1 M HCl solution (100 ml g^−1^) for 30 minutes at 100 °C with 300 rpm stirring under a reflux system. After treatment, acid-washed zeolites were treated as described in Section 2.2.1.

#### 2.2.5. Trials for fine-tuning the recipe of FAU-to-LEV

Some batch experiments were conducted to determine a suitable FAU-to-LEV recipe with a relatively short synthesis time and a dilution level compatible with the FB setup. All these experiments were performed using CBV 720 (Si/Al = 15) as parent zeolite and different types of OSDAs, such as choline hydroxide, 1-adamantanamine, or DMPOH. Samples were prepared by mixing USY CBV720 (Si/Al = 15, or SiO_2_/Al_2_O_3_ = 30), sodium hydroxide or ammonium fluoride, OSDA, and MilliQ water. The mixture was stirred for 16 h at room temperature and then loaded into 25 ml batch autoclaves with stirring (600 rpm) and placed into the oven. Interzeolite conversion/crystallization was conducted at different molar compositions and synthesis conditions as mentioned in Table S2. After crystallization, the product was treated as detailed in Subsection 2.2.1.

#### 2.2.6. Synthesis of ZnZrO_*x*_

ZnZrO_*x*_ solid solution was prepared by co-precipitation method according to Wang *et al.*^28^ Briefly, 2.55 g of Zn(NO_3_)_2_·6H_2_O and 15 g of ZrO(NO_3_)_2_·*x*H_2_O were dissolved in 300 ml of water for 30 minutes. Next to dissolution, co-precipitation was carried out with dropwise addition of 0.3 M ammonium carbonate solution under vigorous stirring at 70 °C, followed by aging for 3 h. The precipitated solution was centrifuged three times with water and one time with acetone and the resulting solids were dried at 100 °C overnight. Finally, the dried solids were calcined at 500 °C for 3 h.

### 2.3. Characterization techniques

#### 2.3.1. Powder X-ray diffraction (PXRD)

The structure and crystallinity of the as-made (uncalcined) zeolites were examined with powder X-ray diffraction (PXRD) using a high-throughput STOE STADI P Combi diffractometer in transmission mode with focusing Ge (111) monochromatic X-ray incident beams (*λ* = 1.5406 Å, Cu Kα source). The scanning time for the powder of each sample was 10 min.

#### 2.3.2. Nitrogen physisorption

The porosity of calcined zeolites was measured by nitrogen physisorption on a TriStar II 3020 Micrometrics instrument at −196 °C in the relative nitrogen pressure (*P*/*P*_0_) range of 0.01 and 0.99. The *t*-plot method (Harkins and Jura)^29^ is used on the adsorption branch to determine micropore volumes.

#### 2.3.3. ICP-AES

ICP-AES analyses were conducted on PerkinElmer Optima 3300 DV. Samples for the analysis were prepared *via* the procedure called “HF method”. Before an ICP-AES measurement, 50 mg of each sample was dissolved using 2 ml of HF and 0.5 ml of aqua regia. After 3 h, they were neutralized using 33 ml of boric acid solution (30 g l^−1^) and diluted with Milli-Q water. After an additional 2 h, samples were diluted using 0.42 M HNO_3_ in water by *a* factor of 26. *Caution: handling HF requires strict safety measures and should only be undertaken by trained personnel.*

#### 2.3.4. Thermogravimetric analysis (TGA)

The organic content of all as-synthesized materials was determined by thermogravimetric analysis (TGA) under air using a TGA Q500 from TA Instruments. The heating rate was set at 10 °C min^−1^ up to 800 °C.

#### 2.3.5. Fourier transform infrared spectroscopy (FTIR)

FTIR was performed using Nicolet iS20 equipped with a deuterated triglycine sulfate (DTGS) detector. Prior to analysis, samples were pressed into precisely weighed self-supported wafers of 5–15 mg cm^−2^ and degassed *in situ* at 400 °C (5 °C min^−1^ heating rate) for 6 h under vacuum (<1 mbar). After degassing, the cell was cooled to 150 °C, and a reference spectrum of the material was recorded with an accumulation of 64 scans at a resolution of 4 cm^−1^. The spectra were normalized against the density of the pellets for the analysis (∼10 mg cm^−2^ of dry zeolite).

For the methanol adsorption experiments, methanol vapor (MeOH) pulses of around 40 mbar each were introduced *in situ* onto the activated zeolite samples. MeOH adsorption was followed at 150 °C until saturation (saturation = no difference between two consecutive measured spectra). Upon saturation, the gradual thermal desorption of MeOH was carried out under vacuum (<0.1 mbar) with a 50 °C step (150, 200, 250, 300, and 350 °C) and an isothermal hold of 60 or 30 minutes at each temperature. A spectrum was registered for each desorption step. The MeOH adsorption and desorption spectra were recorded with an accumulation of 128 scans at a resolution of 2 cm^−1^. For better comparison among the different samples, the spectra were normalized to the density of the pellets.

#### 2.3.6. ^27^Al and ^29^Si solid-state nuclei magnetic resonance (NMR)

Solid-state NMR measurements were performed with a Varian Inova spectrometer and an 11.74 T Oxford magnet, thus 500 MHz for protons, using a 4 mm Chemagnetics solids probe. The powdered samples were packed in 4 mm zirconia rotors and spun at 15 kHz at the magic angle. A *π*/2 acquisition pulse sequence was used for all of experiments. ^27^Al spectra were referenced to Al(NO_3_)_3_ solution at 0 ppm, while ^29^Si spectra were referenced to tetrakis(trimethylsilyl)silane (TKS) at −9.9 ppm.

#### 2.3.7. Ammonia temperature programmed desorption (NH_3_-TPD)

NH_3_-TPD was performed with an Altamira AMI-300 instrument connected to a thermal conductivity detector (TCD) to analyze the products. 30 mg of each sample was measured and placed in a U-tube quartz reactor. In general, a sample was first treated for 1 h under the flow of 25 ml min^−1^ of pure He from 25 °C to 400 °C, followed by cooling to 30 °C under the flow of He. Next, its surface was saturated by 25 ml min^−1^ flow of 5 vol% NH_3_/He from 30 to 100 °C at a ramp rate of 1 °C min^−1^ and held for 1 h. Finally, the desorption of NH_3_ occurs under the flow of 25 ml min^−1^ of He from 100 °C to 700 °C. An online mass spectrometer (MS) was also connected to the system to monitor different species by tracking various *m*/*z* values (*m*/*z* = 14, 15, 16, 17, 18).

### 2.4. Catalytic test

The catalytic tests were performed in a fixed-bed reactor (PID Eng&Tech) with feed preheating (at 140 °C). A total amount of 150 mg of the granular mixture of metal oxide (ZnZrO_*x*_) and zeolite (each with a particle size of 125–250 µm) in the fixed ratio of 2 : 1 (so 50 mg zeolite) was loaded into the reactor-fitting inert tube (I.D. of 5 mm) in a mixed bed configuration. ZnZrO_*x*_ was synthesized by the co-precipitation method following the procedure reported by Wang and coworkers.^28^ All catalysts were activated under the pure N_2_ flow of 50 ml min^−1^ at *T* = 380 °C (with a heating ramp of 10 °C min^−1^) and *P* = 4.0 MPa for 1 h prior to the reaction. The reactants N_2_, CO_2_, and H_2_ at a fixed composition of 12/22/66 V/V/V% were fed to reach space velocities of 18 000 ml g_cat_^−1^ h^−1^, and the reaction was carried out at *T* = 380 °C and *P* = 4.0 MPa. The reaction products were analyzed by an online GC. The permanent gases (H_2_, CO_2_, CO, and N_2_) were detected by two thermal conductivity detector (TCD) channels, either using He as a carrier gas or using Ar (for H_2_). Hydrocarbons were detected by a flame ionization detector (FID). CO_2_ conversion (*X*_CO_2__), product selectivity (*S*_*i*_), and product space time yield (STY_i_) (*i* = MeOH (methanol), CO (carbon monoxide), DME (dimethyl ether), hydrocarbons (HCs)) were calculated as shown in eqn (1)–(3). Finally, the steady-state averaged values of the performance descriptors were reported during time on stream (TOS) of 2–5 h.1
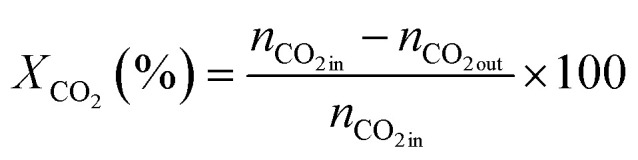
2
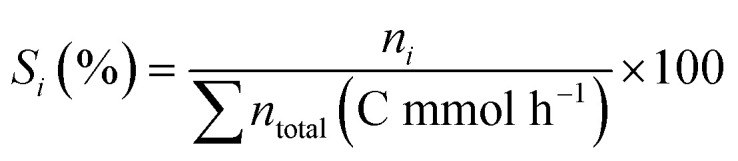
3
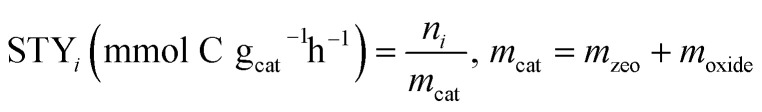


## 3. Results and discussion

### 3.1. Tracking and manipulating interzeolite conversions

As mentioned in the introduction, in the IZC, there is a “transient window” that can be utilized to manipulate the crystallization process, here with the aim of obtaining zeolite catalysts with higher silica content. Previously, the FB platform has shown promising utility in *in situ* monitoring of zeolite formation.^11^ Following this approach, we initially time-mapped both IZCs of FAU-to-CHA and FAU-to-LEV systems in the FB reactor, then designed and performed a timed addition of silica during synthesis, and these cases are presented separately below.

#### 3.1.1 FAU-to-CHA

Fig. S1 illustrates the evaluation of the IZC during its early stages tracked *via* the FB reactor. 30 minutes after the initiation of the synthesis, some reflections associated with the FAU phase (the parent zeolite) are still present. However, after 1 hour, no major reflection of either FAU or CHA phase could be detected (neither in the parent nor the daughter zeolite). CHA reflections start to appear after 1.5 hours, indicating that the first hour after the start of synthesis can be classified as a transient window. This aligns with other reports regarding the siliceous IZC (Si/Al > 35) kinetics of FAU-to-CHA (same starting precursors with slightly lower water content in the starting composition), where a transitional phase occurs approximately between 30 minutes and 1 hour.^23^ During this period, the species could be the remnants of the dissolving starting zeolite, which no longer exhibits long-range order detectable by X-ray diffraction and has not yet converted to a daughter zeolite form. Based on the time-mapping data, further experiments were conducted in the FB reactor while an additional silica source was fed during synthesis. Following the 1 hour transient window, a fixed volume of 25 ml colloidal silica solution was fed into the reactor in all experiments, with varying feeding durations of 1, 5, 13, and 20 hours. It should be noted that dilute sources should be used in the FB platform, as more concentrated feeding solutions (*e.g.*, >10 wt%) might block the feeding lines of the reactor.

**Fig. 2:**
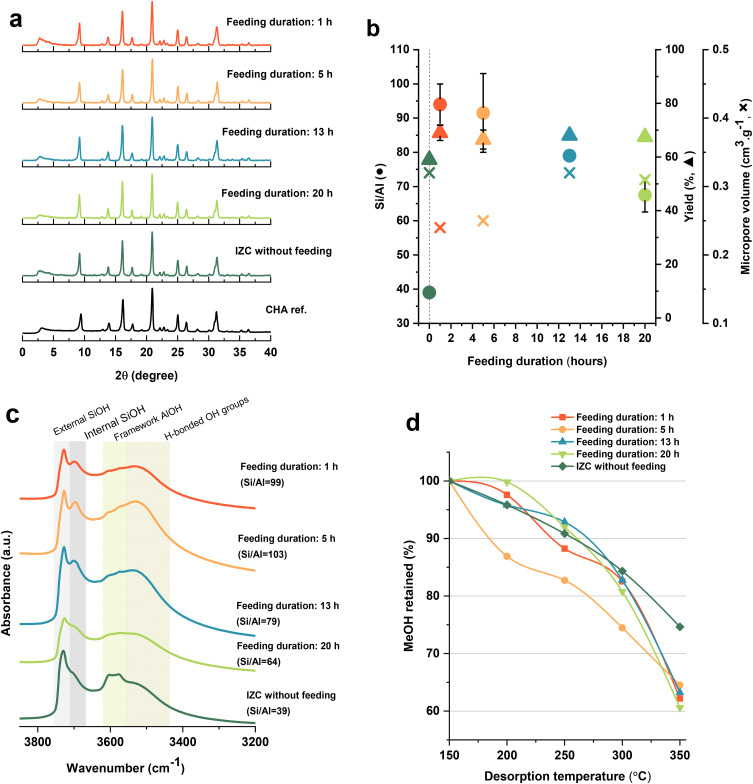
Characterization data for zeolites and references made with starting molar composition as follows: 1 SiO_2_: 0.025 AlO_2_^−^: 0.35 TMAdaOH: 25H_2_O, and the final molar composition (after feeding) was: 1 SiO_2_: 0.012 AlO_2_^−^: 0.17 TMAdaOH: 27.47H_2_O. (a) PXRD; (b) Si/Al ratio (●), yield (▲), and micropore volume (×) [the 0 h feeding duration is IZC without feeding]; and (c) FTIR spectra of the FB-made CHA zeolites obtained with different feeding durations (*e.g.*, 1, 5, 13, and 20 h) in comparison to the classic IZC batch sample. Error bars are a result of some points being reproduced once to examine the repeatability of the obtained results. (d) Relative amount of MeOH retained (% of adsorbed amount) at different desorption temperatures. Lines were added as a guide to the eye. Note that the plot starts at 55% for the *y*-axis.

All FB-made zeolites were fully crystallized after 24 hours, regardless of feeding duration, and there was no sign of impurities or a separate phase in XRD spectra (Fig. 2a). Intermediate feeding during the “transient window” plays a significant role here, as the introduction of colloidal silica at time zero, prior to the dissolution step in the IZC method, can considerately disrupt the system, increasing the risk of forming a secondary, undesired layered silicate phase alongside the target zeolite (Fig. S2). Thus, with well-designed intermediate addition, this problem can be overcome, aiming for a pure-phase CHA.

N_2_ adsorption isotherms and TGA curves of these FB-made zeolites are also shown in Fig. S3 and S4, respectively. Although TGA curves appear similar, there is a difference in N_2_ adsorption capacity between samples with different feeding durations. CHA zeolites synthesized with longer feeding durations (*e.g.*, 13 h and 20 h) exhibited higher adsorption capacities (all type I, microporosity of 0.31 cm^3^ g^−1^), while shorter feeding (*e.g.*, 1 h and 5 h) resulted in samples with lower amount of the adsorbate (micropore 0.24 cm^3^ g^−1^). This observation can be rationalized by considering the role of supersaturation in zeolite formation. Longer feeding times, corresponding to a slow addition of aqueous silica solution, would not significantly affect the supersaturation level in the synthesis medium, especially during critical moments such as nucleation and onset of crystallization. Therefore, these conditions favor controlled crystal growth, facilitating proper framework ordering and minimizing the formation of defects (also demonstrated later by FTIR and NMR). Following this hypothesis, the resulting zeolite structures have a higher micropore volume (also plotted in Fig. 2b). In contrast, short feeding times, which introduce the aqueous silica source rapidly, would also dilute the synthesis medium, greatly affect the supersaturation level, and theoretically disrupt the system and sacrifice some of the micropores in the final product by prolonging the overall crystallization time (Table S1).

Yield and Si/Al_ICP_ (final calcined material) ratio of FB-made CHA zeolites are represented in Fig. 2b. The yield of all FB-made CHA zeolites (except IZC without feeding) is around 70% and is not affected by different feeding durations (the amount of added silica was taken into account). As mentioned earlier, in a standard IZC conversion of FAU-to-CHA, the final Si/Al ratio complies with that of the parent zeolite; as can be seen, it is approximately 40, which is the same as the starting FAU zeolite. On the other hand, all FB-made samples, with intermediate feeding of Si, resulted in an increased Si/Al ratio in the final product, and interestingly, the feeding duration (feeding rate) plays a key role here. Samples synthesized with short feeding durations (faster addition) exhibit higher Si/Al ratios (even ≥100), whereas those prepared with longer (slower) silica addition show lower Si/Al ratios. A likely reason for this is the formation of siliceous species, known as “internal silanols,” in FB-made zeolites at higher feeding rates, which will be thoroughly discussed later using FTIR and TPD techniques.

In separate experiments, a different cooling approach was used. Instead of immediate cooling with ice, the reactor was allowed to cool gradually, with the temperature decreasing slowly from 160 °C to room temperature over a period (timed-cooling). A brief discussion is presented in Section S2.

To further elaborate on these materials and understand the Si/Al and yield trends, an extensive characterization was performed using FTIR (also with probe molecule adsorption–desorption) and Solid-State NMR. Our FTIR analysis of FB-made samples' spectra (Fig. 2c) in the OH vibrations region, reveals four main features: (1) a sharp peak could be observed for all samples around 3725 cm^−1^ which is assigned to external silanol groups; (2) there is a distinct peak for some FB-made samples (Si/Al >60) around 3700 cm^−1^ assigned to internal silanol groups. However, its intensity is very low for both the classic IZC and the sample with 20 hours of feeding duration; (3) there is a broad peak (∼3500 cm^−1^) assigned to H-bonded OH groups in FTIR spectra of samples with feeding durations of 1, 5, and 13 hours (Si/Al of 99, 103, and 79, respectively), indicating the defect-rich frameworks of these zeolites. Band assignment was performed according to Bordiga *et al.*^30^ These observations suggest that the very slow feeding rate (20 µl min^−1^) promotes a more ordered and defect-free framework, alike the batch IZC. On the other hand, a faster feeding rate will increase the possibility of Si species acting as siliceous domains, resulting in a defect-rich lattice; (4) as for the framework Al–OH species (around 3600 cm^−1^), as expected, the intensity of this band decreases with the increase in the Si/Al ratio, reflecting less Al in the zeolite framework. However, contrary to this hypothesis of high local silica concentration is the fact that these syntheses are stirred (top down) at 900 rpm, meaning that local gradients can only exist for short periods in our setup, except when one would consider a local drop falling into the mixture and being incorporated rapidly without mixing (unlikely, and moreover, the drops also contain 90 wt% of water).

Since the reactivity of these zeolites will be tested in the conversion of CO_2_ to olefin (CTO), MeOH adsorption and retention capacity were performed as described by Sajid and coworkers.^31^ They described this method for calculating MeOH adsorption and retention capacity based on the integrated area of the FT-IR spectral bands (in the region of 2800–3000 cm^−1^). The MeOH adsorption capacity of FB-made zeolites is shown in Fig. S6. It demonstrates that samples with feeding durations of 1, 5, and 13 hours exhibit a lower adsorption capacity for MeOH, which might be due to their defect-rich frameworks, evidenced earlier with FTIR, but also to the pore volumes. However, many parameters (especially void space) affect adsorption, which could explain why the 13 h feeding samples exhibited low MeOH adsorption while having a high physisorption capacity (0.32 cm^3^ g^−1^). After desorption at 150 °C, the physisorbed methanol was removed, leaving only the chemisorbed fraction. The residual chemisorbed methanol amount was then used to quantify methanol retention at higher temperatures relatively: a retention of 100% at 150 °C corresponds to the total chemisorbed methanol remaining. Overall, all FB-made samples, synthesized *via* feeding, exhibited around 60–65% MeOH retention at the highest desorption temperature (350 °C, Fig. 2d). In comparison, the standard IZC sample showed around 75% MeOH retention at the same point. The sample with a 5 h feeding duration stands out with the lowest retention capacity among all samples; this limited retention capacity may affect product selectivity in methanol to hydrocarbons due to limited contact time. The MeOH retention capacity graph *vs.* Si/Al ratio of samples is also depicted in Fig. S7 for a clear quantitative comparison. The interaction of methanol with FB-made samples is shown in Fig. S8 by comparing the FTIR spectra collected immediately after sample activation at 400 °C with those obtained after methanol desorption at 150 °C and 350 °C. An insignificant decrease in the FTIR spectra was observed at desorption temperatures of 150 °C and 350 °C.

**Fig. 3:**
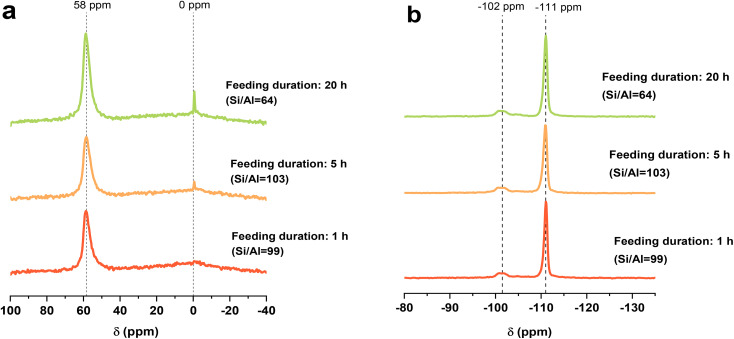
Solid-state NMR spectra for (a) ^27^Al and (b) ^29^Si of FB-made CHA zeolites with different feeding durations.

^29^Si NMR spectra in Fig. 3 show two distinct signals: a sharp peak around −111 ppm assigned to Q^4^(0Al) species (silicon bonded to 4 silicon atoms with no Al neighbors) and a smaller double peak around −102 ppm associated with Q^3^ species (different types of defect sites). Generally, there is a signal in between which is assigned to Q^4^ species with one or more Al neighbors, but here it couldn't be detected. It might be due to the lower amount of these species compared to Q^4^(0Al) species, making them less detectable because of the sensitivity level of this analysis. On the contrary, signals related to these Q^4^ species could be detected in NMR spectra of the classic IZC sample.^4,27,32^Al NMR spectra (Fig. 3) of samples show two distinct signals: a sharp peak around 58 ppm associated with tetrahedrally coordinated Al species in the framework and a relatively smaller peak around 0 ppm assigned to (extra-framework) octahedral aluminum. Interestingly, there is a trend of having more extra-framework Al species when using a longer feeding duration (slower feeding rate). On the other hand, this extra-framework Al signal could not be observed in the classic IZC sample, implying a well-ordered framework with fewer extra-framework species.^4,32^ This difference might be due to the prolonged feeding process (20 hours), which could prevent some Al species from being inserted into the framework. That might be the reason for the sharp peak of extra-framework aluminum species in ^27^Al-NMR. These signal assignments were performed according to Engelhardt.^33^

In addition to the NMR results and the previously discussed FTIR data, there is further evidence that supports the presence of a high concentration of silanol defects in the FB-made zeolites (especially those with shorter feeding durations). The sample with the highest Si/Al ratio (Si/Al = 103) was tested using NH_3_-TPD, during which the TCD signal was recorded alongside mass spectrometry (MS) analysis. As shown in Fig. S9a, the TCD signal exhibits an unusual increase above 500 °C compared to the counterpart of classic IZC (without feeding Si/Al = 39). Simultaneously, the mass spectrometry data indicate a rising release of H_2_O (Fig. S9b). This behavior is likely attributed to the collapse or condensation of hydrophilic silanol groups at elevated temperatures.^34,35^

#### 3.1.2 FAU-to-LEV

The FAU-to-LEV system was also studied to evaluate how effective the FB approach generally is for achieving higher Si/Al ratios. LEV is another cage-containing zeolite (same as CHA) which has the 8-membered rings in a 2-dimensional arrangement, compared to the 3-dimensional arrangement of CHA.^36^ Thus, a different catalytic behavior could be expected due to different framework dimensionality. These structural features of LEV suggest a high potential for a specific shape selectivity for light olefins production. Notably, it was found that the Si/Al ratio of LEV influences the product distribution, with higher-Si LEV producing more light olefins.^27^

To start with, we have performed extensive trials to fine-tune the IZC recipe using different OSDAs (see Section S3 and Table S2) and were able to achieve a pure LEV phase using DMPOH in 2 days. In short, Fig. S10 illustrates the adjusted transformation of FAU-to-LEV during its early stages using the sampling mode in the FB reactor. 30 minutes after the initiation of the synthesis, no reflections associated with the FAU phase could be detected in the PXRD spectrum. Moreover, samples taken after 1 and 2 hours still do not show either parent or daughter reflections. LEV reflections start to appear after 3 hours, indicating that the period of 30 minutes to 2 hours after the start of synthesis can be classified as a transient window. Similar to the previous section, further experiments with intermediate addition were performed in the FB reactor, while a fixed volume of diluted silica source (15 ml) was fed into the reactor using different feeding durations of 1, 5, and 12 hours.

**Fig. 4:**
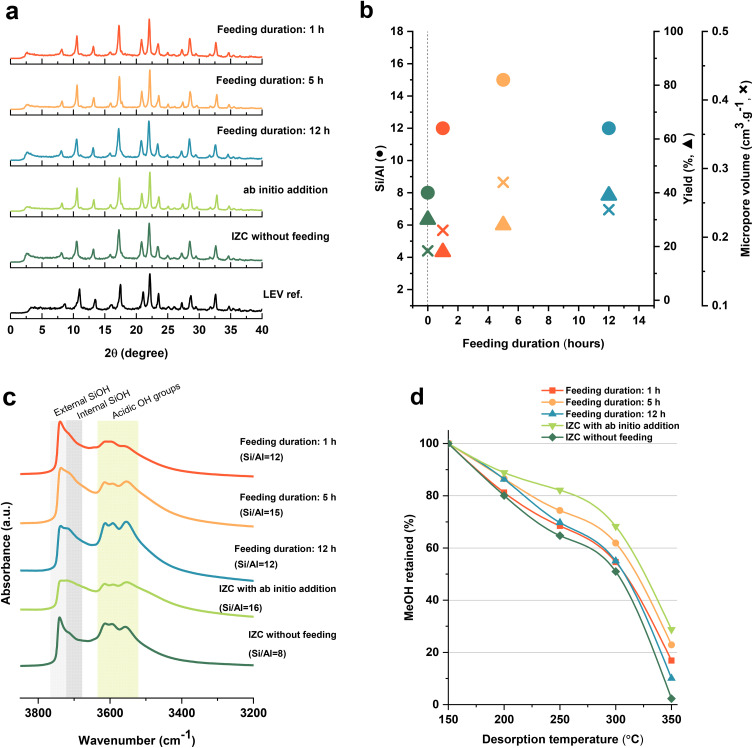
Characterization data for zeolites and references made with starting molar composition as follows: 1 SiO_2_: 0.066 AlO_2_^−^: 0.2 DMPOH: 0.3 NaOH: 10H_2_O, and the final molar composition (after feeding) was: 1 SiO_2_: 0.045 AlO_2_^−^: 0.13 DMPOH: 0.2 NaOH: 16.6H_2_O. (a) PXRD; (b) Si/Al ratio (●), yield (▲), and micropore volume (×) [the 0 h feeding duration is IZC without feeding]; and (c) FTIR spectra of the FB-made LEV zeolites obtained with different feeding durations (*e.g.*, 1, 5, and 12 h) in comparison to the sample with *ab initio* addition and classic IZC batch sample; (d) relative MeOH retained (% of adsorbed amount) over FB-made samples at different desorption temperatures. Lines were added as a guide to the eye.

All FB-made zeolites were fully crystallized after 48 hours, regardless of feeding duration, and there was no sign of impurity or separate phase in XRD spectra (Fig. 4a). Moreover, *ab initio* addition of colloidal silica also resulted in pure LEV phase. In contrast to the FAU-to-CHA system, the introduction of Si here didn't disrupt the system, and there was no secondary phase alongside the target zeolite. It is possibly related to the dissolution step of different IZC systems (with different conditions and OSDA), especially noticeable in the pH value. To study the difference, pH measurements were run on the room temperature systems in the aging phase: in the FAU-to-CHA case (the starting composition before Si addition, but after 16 hours of aging) the pH remains almost unchanged (∼13.7), whereas in the FAU-to-LEV case the pH dropped to 12.3 upon aging, which means that the dissolution of FAU has already started (confirmed by Fig. S10). Subsequently, the introduction of an aqueous Si solution, which increases the water content and changes the synthesis environment, would not significantly impact the dissolution step that has already started. Comparing Fig. S1 and S10 reveals that this step occurs faster in the FAU-to-LEV than for the FAU-to-CHA system: in the CHA system, some FAU reflections can still be detected after 30 minutes, whereas there is no sign of the parent zeolite after 30 minutes in the LEV system. This is mostly due to the more basic medium in the FAU-to-LEV system, 0.05 mol OH^−^/H_2_O compared to the 0.014 mol OH^−^/H_2_O from the other system. The difference in the transition window represents only a part of the broader effects introduced by using different OSDAs, which impact the stabilization and formation of distinct intermediate phases. However, a full mechanistic analysis is beyond the scope of this study.

N_2_ adsorption isotherms and TGA curves of these FB-made zeolites are also shown in Fig. S11 and S12, respectively. Similar to the previous section, the TGA curves for FB-made samples seem alike; however, a noticeable (and similar) trend is observed in the N_2_ adsorption capacities. LEV zeolites synthesized with longer feeding durations of 5 and 12 hours exhibited higher adsorption capacities, while the shortest feeding (*e.g.*, 1 h) resulted in samples with the lowest amount of the adsorbate (also true for the *ab initio* sample). Introducing the aqueous silica source rapidly within a short time (*e.g.*, 1 h) seems to result in a sample with relatively lower microporosity [similar to the CHA system] (Table S3). As discussed earlier, we tentatively attributed this to the more sudden disruptions in the synthesis medium and the supersaturation level. However, surprisingly, 5 hours of feeding duration resulted in a more well-ordered porous structure (higher micropore volume) than a 12 hours feeding duration in LEV synthesis, which means that a longer feeding duration does not always lead to higher microporosity (*e.g.*, as seen in Section 3.1.1). Lastly, it should be mentioned that all of the FB samples produced with timed addition possess higher microporosity (0.21–0.28 cm^3^ g^−1^) than classic IZC (0.18 cm^3^ g^−1^). On the other hand, *ab initio* addition of Si resulted in a lower micropore volume (0.17 cm^3^ g^−1^), which could be attributed to interference with the critical synthesis steps (*e.g.*, dissolution) and raising the chance of having amorphous species. The reported range of micropore volumes for LEV zeolites synthesized with DMPOH is between 0.19 and 0.24 cm^3^ g^−1^.^37^

Yield and Si/Al_ICP_ (final material) ratio of FB-made LEV zeolites are represented in Fig. 4b. The yield of all FB-made zeolites (except IZC without feeding) is preserved between 30-40% and is slightly affected by different feeding durations (the amount of added silica was taken into account). In cases where silica is added *ab initio*, the synthesis yield drops sharply to 16%, indicating that the introduced species disrupt the crystallization pathway and extend the synthesis time. For the classic interzeolite conversion of FAU-to-LEV (without extra silica addition), the final Si/Al ratio was slightly lower than that of the parent zeolite: Si/Al 8 *vs.* 15, respectively (similar behavior for LEV crystallization system has been documented before^37^). All FB-made samples, with intermediate feeding of Si, resulted in an increased Si/Al ratio in the final product (*vs.* the reference): 12, 15, and 12 in the case of 1, 5, and 12 hours of feeding, respectively. It can be observed that feeding duration greatly affects yield but does not significantly influence the Si/Al ratio. It should be noted that the sample with *ab initio* addition of Si showed a Si/Al ratio of 16. Thus, although both the *ab initio* addition of Si in batch mode and feeding using the FB platform resulted in similar Si/Al ratio, the FB approach seems to offer an advantage when taking into account the higher synthesis yield and micropore volume, likely because an interference with the synthesis pathway is minimized, and therefore serves as an example highlighting the importance of timed addition. A brief discussion about the amount of added Si solution and the final Si/Al ratio in the FB platform can be found in Section S4.

FTIR spectra of FB-made samples (Fig. 4c, FTIR OH vibrations region) show three main regions of interest: (1) the peak around 3740 cm^−1^ was assigned to the external silanol group; (2) a peak shoulder around 3730 cm^−1^ is still present and is assigned to internal silanol groups; (3) three peaks at 3616, 3593, and 3554 cm^−1^, all representative of different framework Al–OH groups. These three peaks correspond to the three different sites of Al in the framework. These band assignments are according to the literature on LEV zeolites.^27,37^ Still, there is a notable lack of spectroscopic studies, particularly on LEV zeolites. However, samples with *ab initio* addition or a short feeding time (1 hour) exhibited a similar pattern and fewer acidic OH groups, which means that interrupting the synthesis medium in the earlier stages of the synthesis affects the formation of these groups. Nevertheless, samples without feeding or with a long feeding time (*e.g.*, 12 hours) do not significantly affect the synthesis medium, and more of the mentioned acidic groups could be observed in their IR spectra.

Additionally, FTIR with MeOH probe was performed, and the MeOH adsorption capacity of FB-made LEV zeolites is shown in Fig. S14. As it should be expected, samples prepared with a long feeding duration (12 h) or without feeding exhibit higher MeOH adsorption capacity, as prolonged feeding causes minimal disruption and produces a well-ordered structure similar to standard IZC. Moreover, the sample with *ab initio* addition showed even lower adsorption capacity than the FB samples and aligns with our observations about reduced micropore volume and the possibility of having amorphous species. Fig. 4d shows the retained MeOH based on different desorption temperatures. The MeOH retention behavior closely follows the Si/Al ratio; samples with higher Si/Al ratio exhibited greater retention capacity (even at 350 °C). However, as discussed in the previous section, for MeOH adsorption, “defects” in the structure, micropore volume, and existence of amorphous species are the key factors; however, for retention, it may be related to acidity. Furthermore, Sajid and coworkers conducted steam treatment on small-pore zeolites and found that differences in MeOH retention are caused by variations in acidity.^31^

Overall, both FAU-to-CHA and FAU-to-LEV systems were manipulated using a timed addition approach in FB reactors, and pure zeolites with an elevated Si/Al ratio were obtained. The FB approach overcame the issue associated with the *ab initio* addition of Si species and yielded unique high-Si CHA zeolites (Si/Al > 90), which exhibited interesting features (*e.g.*, internal silanols) and showed different behavior in MeOH desorption (less retention), making them interesting for catalysis. LEV systems mirrored this behavior to some extent, but the final Si/Al values obtained do not lie outside of the range which one can easily make in OH^−^ media with tuned batch recipes. Literature shows that the Si/Al ratio of parent zeolite (FAU) and the type of OSDA have a significant effect on the flexibility of the LEV synthesis system toward high Si/Al products. For instance, making LEV zeolites with a Si/Al ratio of 30 is feasible in hydroxide media by using dealuminated FAU (Si/Al = 107) as the parent zeolite.^37^ OSDAs are important in FAU-to-CHA and LEV systems. For DMP (with C/N: 7), the charge density is higher than for TMAda (with C/N: 13). One could use this to rationalize Si/Al difference in the output, but in fact, when each large cage of either CHA or LEV incorporates one OSDA molecule (often the case and the numbers from TGA do suggest this), and no other charge compensation is present (so no Na^+^ compensating framework aluminum), the Si/Al ratio of LEV should be 8 and that of CHA 11. However, when going to larger Si/Al ratios in hydroxide media, the OSDA charge needs to be compensated by negatively charged defects (often SiO^−^). It is our hypothesis that for certain structures where the OSDA fits into the cage very well, it is easier for a structure to allow for SiO^−^ based compensation and thus increased Si/Al ratios. For LEV, the fit of DMP with the cage is less tight and this might counteract increasing Si/Al ratio in the product. Whether this is a thermodynamic explanation (based on charge stabilization and defect-OSDA balancing energetics) or a kinetic one (based on the ability of a framework to grow around or with an OSDA while incorporating a defect) is hard to deconvolute now.

### 3.2. Catalytic evaluation of high-silica zeolites in CO_2_ hydrogenation

To explore the potential application of the synthesized samples and assess the anticipated benefits of high Si/Al zeolites prepared in hydroxide media, some FB-made CHA and LEV zeolites were tested in the CO_2_ hydrogenation reaction coupled with methanol upgrading, and compared with their classic IZC counterparts (batch mode). In CO_2_ hydrogenation to hydrocarbons process, CO_2_ is first converted to methanol over metal oxide catalyst (*e.g.*, ZnZrO_*x*_), and then the intermediate product (oxygenates) undergoes further conversion to paraffins/olefins over zeolites (*i.e.*, tandem catalytic system).^38,39^ The ZnZrO_*x*_ used here is a well-known reference methanol synthesis catalyst (XRD pattern see Fig. S19) and a test without zeolite addition shows its expected behavior^31^ (Fig. S18): high space time yields of methanol are observed until 360 °C, after which CO takes over due to reverse water gas shift or methanol decomposition catalysis. By adding the zeolites, the sum of the space time yields of methanol and methanol-mediated products (olefins and paraffins), at 380 °C, is increasing to a range between 12 and 15 mmol g_cat_^−1^ h^−1^ in comparison to the sole ZnZrO_*x*_ system (Fig. S18), where a maximum STY of MeOH at 380 °C was 8.5 mmol g_cat_^−1^ h^−1^ (and CO is the dominant product). This differentiates the performance of ZnZrO_*x*_ and zeolite and evidences the role of coupling in line with previous reports.^31,40^

**Fig. 5:**
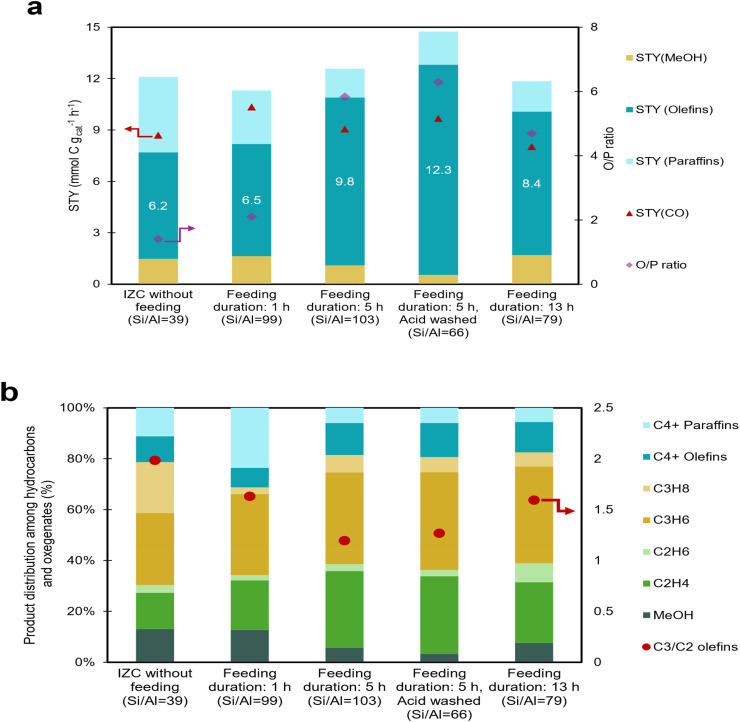
(a) Steady state averaged (TOS = 2–5 h) Olefin to Paraffin ratio (O/P) and product space-time yield (STY, mmol C g_cat_^−1^ h^−1^) for DME, methanol (MeOH), CO, olefins, and paraffins, (b) product distribution among hydrocarbons and oxygenates over FB-made CHA zeolites. Note that DME production was not detected. Reaction conditions: *T* = 380 °C, *P* = 4.0 MPa, GHSV = 18 000 ml g_cat_^−1^ h^−1^, H_2_/CO_2_ = 3.0.

In Fig. 5, the performance of FB-made CHA samples achieved *via* different feeding durations is compared with classic IZC CHA. To support this, CO_2_ conversion, product space-time yield (STY), and olefin to paraffin ratio (O/P) as a function of time on stream (TOS) were also plotted and depicted in Fig. S15. A similar CO_2_ conversion trend was observed for CHA samples, while significant differences in product space-time yields were noted. Compared to other samples, the classic IZC sample without feeding (Si/Al = 39) showed an O/P ratio of approximately 1.5 and an olefin yield of 6.2 mmol C g_cat_^−1^ h^−1^. Its higher paraffin production correlates well with the MeOH-IR data and its high retention capacity. On the contrary, the FB-made samples (Si/Al = 79, 99, and 103) showed a higher O/P ratio and lower paraffin yield. For instance, the FB-made sample with 5 hours of feeding (Si/Al = 103) showed a remarkable catalytic performance, achieving the highest O/P ratio of 5.8 and olefin yield of 9.8 mmol C g_cat_^−1^ h^−1^ relatively. Again, these results align with the MeOH-IR analysis from Section 3.1.1, which indicated a relatively lower methanol retention capacity for this high-silica sample (Fig. 2d). From a catalytic perspective, this lower retention capacity can be indicative of a shorter residence time for oxygenate species in catalytic conditions, or fewer oxygenate species adsorbed on zeolite active sites, and consequently, a lower chance of secondary hydrogenation or oligomerization, thus suppressing paraffin formation.^31,41^ Robijns and coworkers, in our group, reported a similar observation^4^ in their split synthesis; they achieved a high-silica CHA zeolite with a Si/Al ratio of 111 and benchmarked it against a CHA synthesized *via* standard IZC (Si/Al = 15). They reported that the O/P ratios increased from 0.5 in the standard sample to 2.6 in the split-synthesized sample, as well as an olefin yield of 10 mmol C g_cat_^−1^ h^−1^ during reaction at 380 °C.

It should be noted that the sample with 1 hour of feeding produced 6.5 mmol C g_cat_^−1^ h^−1^ olefins and showed an O/P ratio of 2 (close to that of IZC without feeding), despite having an elevated Si/Al ratio of 99. However, the sample with 13 hours of feeding showed higher O/P (4.7) and higher olefin yield (8.4 mmol C g_cat_^−1^ h^−1^) despite having a lower Si/Al compared to the sample with 1 hour of feeding. It suggests that the Si/Al ratio is not the only factor involved, and further investigation is needed to identify the underlying factors (partly done below). Fig. 5b depicts the product distribution among the hydrocarbons and oxygenates. The selectivity (among hydrocarbons and oxygenates; so CO-free) to light olefins was 40% for IZC without feeding, increasing to 50, 64, and 60% for samples fed for 1, 5, and 13 hours, respectively. Previous studies have shown that increasing the Si/Al ratio of CHA zeolites can enhance propylene production, a finding that has also been observed by our group previously.^20^ However, in this case, raising the Si/Al ratio resulted in a significantly larger increase in ethylene selectivity.

The propylene to ethylene (*C*_3_^=^/*C*_2_^=^) ratio, a typical indicator of the contribution from the olefinic cycle, is also shown in Fig. 5b. The IZC without feeding showed the highest *C*_3_=/*C*_2_ = ratio of 2, followed by feeding durations of 1 h, 13 h and 5 h. For the sample with a feeding time of 5 h, this ratio stays the same even after acid washing, showing that internal silanols are not the (only or) main factor for the propagation of olefinic cycle. The highest propylene selectivity in the case of the control zeolite without feeding might be attributed to the distribution of framework alumina (*e.g.* paired or isolated) as compared to the other zeolites in this study, as previous studies have indicated the influence of framework pairing on the distribution of products among methanol-derived HCs.^42,43^

Next, as identified by FTIR analysis and discussed in Section 3.1.1, apart from the Si/Al ratio, FB-made zeolites with *in situ* Si addition possess a distinct structural feature not found in the standard IZC sample: the presence of “internal silanols.” These defects were observed in high-silica samples, also reported and appeared to be thermally unstable in TPD upward of 500 °C (Fig. S9b), and this instability is potentially linked to the declining activity (product yield) observed in TOS figures (Fig. S15) for high-silica CHA zeolites. The decrease in activity is solely attributed to the deactivation of the zeolite part as evident from (i) stable CO profile (during TOS = 20 h, Fig. S15) ruling out the deactivation of ZnZrO_*x*_, and (ii) decreased methanol consumption, reducing the equilibrium push toward higher CO_2_ conversion to methanol, resulting in decreased activity.

Moreover, TGA in Fig. S4 of as-made samples shows very similar plots for short or long feeding times, suggesting the siliceous domains are not a large fraction in absolute weight (of the total SiO_2_ content) and do not interact specifically differently toward thermal OSDA decomposition. We attempted to remove these unstable siliceous species through acid washing and examined their correlation with the Si/Al ratio and catalytic activity. High-silica CHA samples (with 1 and 5 hours of feeding duration) were acid-washed as described in Section 2.2.4. This method is preferred for removing internal silanols because alternatives, *e.g.*, silylation, can cause other effects such as blocking the pores.^44^ After acid washing, the Si/Al ratio decreased in both samples (from 99 to 68 and from 103 to 66 in samples with 1 and 5 hours of feeding duration, respectively), the weight of the samples decreased by around 30%, and the intensity of the internal silanol peak was significantly reduced (Fig. S16). In the OH region of these IR spectra, these acid-washed samples exhibit noticeable differences, particularly in their internal silanol defects region (also external *vs.* internal silanol ratio). It can be concluded that a portion of the Si content measured by ICP originates from those siliceous domains and has been washed away. Yet, the acid washing treatment clearly also dissolves part of the silica of the zeolite (desilication, 30 wt% loss). Comparing NH_3_-TPD of the acid-washed samples with the non-washed ones suggests (but with caution) that both acid-washed samples have very similar acidity/strength but still feature a broad signal visible from 500–700 °C (Fig. S20). This signal is less intense than in non-washed samples, suggesting only part of the siliceous species has been removed. Moreover, SEM images of the samples before and after acid washing are shown in Fig. S21.

The sample with a 5 hours feeding duration, which showed the highest O/P ratio and olefin yield, was tested in CO_2_ hydrogenation after being acid-washed (Fig. 5 and S15d). Surprisingly, the olefin yield and O/P ratio increased after acid washing. The acid-washed sample (with Si/Al 66) produced a very high 12.3 mmol C g_cat_^−1^ h^−1^ olefins and showed an O/P ratio of 6.3, suggesting that neither the high bulk Si/Al of the parent sample (103) nor the (high) defect content (Silanols, FT-IR) are the sole main parameters positively affecting olefin production, and it may be due to a combination of subtle but significant differences in defect-related acidic properties of zeolite as well as textural ones such as accessibility and micropore volumes. From the N_2_ physisorption analysis, high-silica CHA samples (with 1 and 5 hours of feeding duration) showed lower micropore volume compared to classic IZC, and this might be attributed to the presence of amorphous species. Even after acid washing, the porosity did not improve due to the partial presence of amorphous species. Moreover, the stability of the zeolite performance on stream was not improved.

Finally, a catalytic trial of FB-made LEV zeolite with a Si/Al ratio of 16 was conducted in CO_2_ hydrogenation (Fig. S17). The results showed that the catalyst was active and stable; however, it did not exhibit considerable selectivity toward olefins (2.6 mmol C g_cat_^−1^ h^−1^) or other byproducts such as DME (2.5 mmol C g_cat_^−1^ h^−1^). While no further studies were conducted due to its limited potential for methanol-mediated CO_2_ valorization, further tuning could enhance its applicability in this reaction (*e.g.*, acid washing^21^).

## 4. Conclusion and outlook

In this work, using the FB platform for zeolite synthesis, we have developed a reactor-based technique to achieve high-silica zeolites. The interzeolite conversions of FAU-to-CHA and FAU-to-LEV were studied as two model systems. Initially, the IZC crystallization profiles were established using the sampling feature of the FB platform. Afterward, by having these profiles as the baseline, manipulation of the synthesis events could be implemented through the feeding feature of the FB platform in order to affect critical stages of material formation. As a result, we were able to produce zeolites with higher Si/Al ratios in a hydroxide medium, which is conventionally challenging (requiring long synthesis time or top-down approaches). Several high-silica CHA (Si/Al > 100) were obtained without prolonging the synthesis. In contrast, the standard IZC route yields the Si/Al ratio of 39, and a commonly encountered upper limit in hydroxide media syntheses is < 60. Interestingly, these tailored siliceous materials exhibit several features, which are not limited exclusively to high Si/Al ratios but also include other improved aspects, such as structural characteristics (*e.g.*, internal silanols) and acidic properties. When less Al is incorporated, the charge of the OSDA has to be stabilized by the growing framework in a different way, and this is likely a charged defect site (interrupted or nest).^34^

As a result, these FB-made zeolites favor highly selective olefin production in CO_2_ hydrogenation (5 h feeding, olefin yield of 9.8 mmol C g_cat_^−1^ h^−1^, with an olefin/paraffin ratio of 5.8). It was found that the Si/Al ratio is not the only factor affecting catalytic activity, but other factors, such as defects,^45^ the presence of amorphous species, and acidity, also play a role. Both defects and amorphous species could reduce the zeolite's stability or performance and cause rapid deactivation. Hence, an acid washing treatment was performed, and those unstable species (detected by FTIR and NH_3_-TPD) and amorphous parts (detected by pore volume measurement) were washed out. Consequently, the acid-washed sample showed a different activity and a higher O/P ratio (6.3) and olefin yield (12.3 mmol C g_cat_^−1^ h^−1^). However, the steady-state zone of olefin production (stable rates) was very short. In the other set of experiments, IZC of the FAU-to-LEV system was investigated, which confirmed the effectiveness of the FB strategy in enhancing the Si/Al ratio within a different small-pore topology. LEV zeolites were obtained with an elevated Si/Al ratio without affecting the yield and pore volume (which occurs in conventional synthesis). These examples highlight the FB strategy as a flexible reactor-based solution, which opens a new pathway toward fine-tuning of zeolite composition and characteristics.

Like any other system, this FB reactor has some limitations: for example, monitoring and tracking are not possible in gel-type synthesis mixtures due to the inability to sample, while feeding highly concentrated solutions is restricted because of the risk of blockage in the feeding line. Dilution degree is important when selecting a system to examine with the FB reactor. For instance, the FAU-to-AEI system is typically synthesized with low water content (*e.g.*, H_2_O/Si around 5), but it needs to be more dilute to be time-mapped *via* FB (sampling). Thus, we tried to increase the water content in the synthesis mixture (*e.g.*, H_2_O/Si around 21 to 40), but we could not synthesize AEI and instead obtained either amorphous material or the ANA phase. Without time-mapping, the FB cannot be used in a targeted way, somewhat limiting it use for low water-content syntheses. On the other hand, the FAU-to-MFI system was very compatible with this reactor, but this system was not investigated in depth, since making high-Si MFI is easy *via* other methods. Another limitation associated with reagent feeding concerns the chemical nature of certain species, such as acids. In theory, no major issues are expected, as the reactor is lined with Teflon, which is resistant to both strongly alkaline and acidic environments, and the peristaltic pump is also compatible with acidic solutions, although it can reduce the operational lifetime. However, the flow of acid through the metallic connector between the pump and the reactor introduces a potential risk of corrosion and we have also noted (not in this study) acidic corrosion of the temperature probe.

Nevertheless, as showcased here, the FB strategy offers unique features, such as tracking synthesis without the cooling-opening method, and can still be applied to different zeolite systems and frameworks by choosing appropriate conditions. Furthermore, the concept of “timed addition” implemented at operational temperature and pressure could be adopted to overcome long-standing challenges in the domain of zeolite synthesis. As part of future research, several challenges could be addressed. For instance, crystallization modifiers can be added to control crystal growth. Moreover, crystallization could be halted at a certain point by the addition of a stopper (*e.g.*, acids). Different zeolite architectural arrangements could also be an interesting topic for FB research. For example, core–shell or intergrowth materials can be explored in FB by the addition of a different array of precursors. In addition, Al or Si zoning might occur by controlling the timing of the addition of silica or alumina precursor solutions. Moreover, intermediate addition of precursors may enable the synthesis of small-pore zeolites with higher Si/Al ratios (such as ERI or KFI) or provide improved control over aluminum distribution within specific topologies, such as CHA. A missing factor in this research is modeling. Modeling zeolite crystallization is very challenging, especially with organics present and the myriad of charges and their solvation effects. Yet, in a world with increasing computational power and machine learning, models for crystallization kinetics (with input of regressed kinetic parameters and *e.g.* data from FB time sampling) could be developed and would certainly provide insights into and complement our intermediate-addition strategy.

## Conflicts of interest

There are no conflicts to declare.

## Supplementary Material

TA-014-D5TA08521G-s001

## Data Availability

The data supporting this article have been included as part of the supplementary information (SI). Supplementary information is available. See DOI: https://doi.org/10.1039/d5ta08521g.
