# Supplementary material for: Reactor-based approach for achieving high-silica zeolites: a fed-batch strategy
Source: J Mater Chem A Mater. 2026 Apr 27;14(33):21799–813. doi: 10.1039/d5ta08521g (PMC13112527; doi:10.1039/d5ta08521g)
Supplement: TA-014-D5TA08521G-s001 [file TA-014-D5TA08521G-s001.pdf]

Supplementary Information (SI) for Journal of Materials Chemistry A.
This journal is © The Royal Society of Chemistry 2026

Electronic Supplementary Information

Reactor-based approach for achieving high silica zeolites: a Fed-batch strategy

Amirhossein Javdani [a], Gleb Ivanushkin [a], Ahmed Sajid [a], Iqtidar Ali Khan [a], Ibrahim Khalil [a], Paola Herrero [a], Quanli Ke [a], Aron Deneyer [a], Michiel Dusselier [a]

[a] *Center for Sustainable Catalysis and Engineering (CSCE), KU Leuven, 3001 Heverlee, Belgium*

michiel.dusselier@kuleuven.be

Section S1. Materials and Chemicals

USY CBV780 [Zeolyst, Si/Al=40]

USY CBV720 [Zeolyst, Si/Al=15]

LUDOX-AS40 colloidal silica [SiO_2, Sigma-Aldrich, 40 wt%]

N,N,N-trimethyl-1-adamantanammonium hydroxide or TMAdamOH [$(CH_3)_3C_{10}H_{15}NOH$, Sachem, 20 wt%]

N,N-dimethylpiperidinium chloride [$C_7H_{16}ClN$,Fluorochem, >98%]

2-Hydroxy-N,N,N-trimethylethanaminium hydroxide or Choline hyroxide [$HOCH_2CH_2N(CH_3)_3OH$, Sigma-Aldrich, 46 wt%]

1-Adamantanamine [$C_{10}H_{17}N$, Thermo Scientific Chemicals, 96%]

Sodium hydroxide [NaOH, Acros Organics, 99%]

Sodium chloride [NaCl, Honeywell Fluca,99%]

Ammonium fluoride [NH_4F, Sigma-Aldrich, >98%]

Hydrochloric acid [HCl, Acros Organics, 37 wt%]

Amberlite™ IRN78 basic Ion Exchange resin [Thermo Scientific Chemicals]

Milli-Q water [18.2 MΩ]

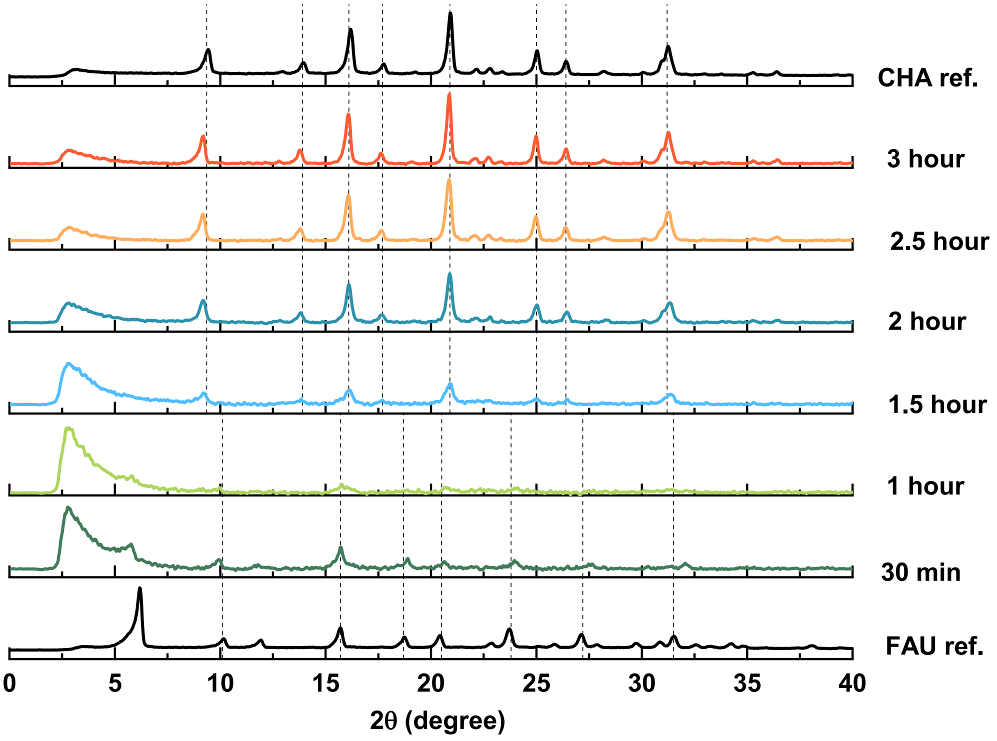

Fig. S1. PXRD diffractograms of samples obtained during early stages of interzeolite conversion of FAU-to-CHA. The synthesis was performed in the FB set-up without feeding ingredients (i.e., batch mode), and sampling was conducted to track the synthesis. The molar composition was used as follows: 1 SiO_2 : 0.025 AlO_2H : 0.35 TMAdaOH : 25 H_2O.

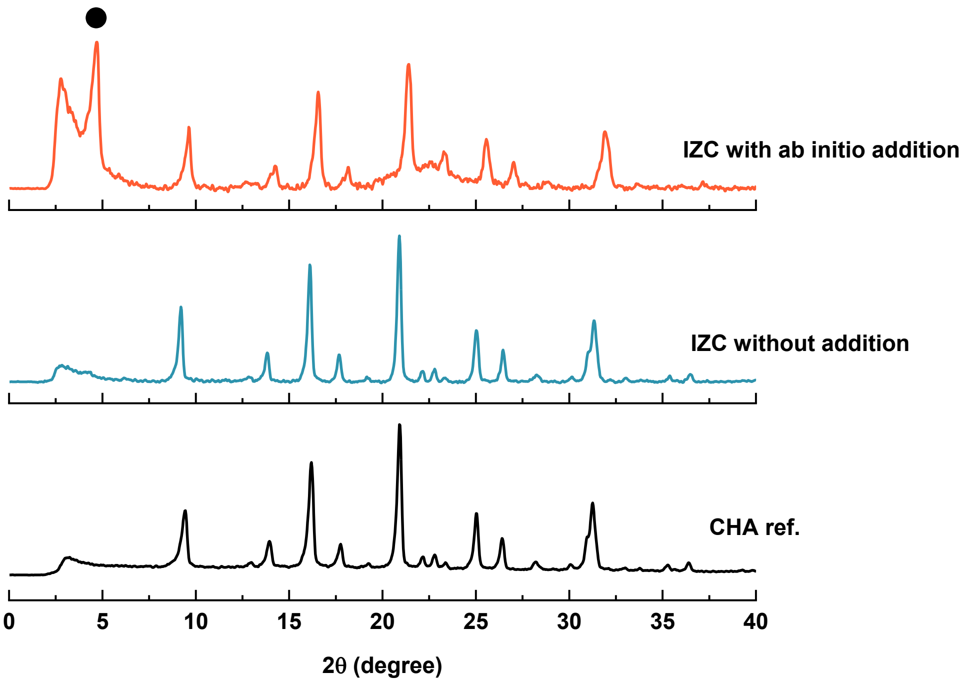

Fig. S2. XRD diffractograms of samples obtained with ab initio addition or without addition of colloidal silica solution during interzeolite conversion of FAU-to-CHA at 160 °C. There is a reflection associated with the layered silica phase marked by ● around 4.5° 2θ in the first spectrum.

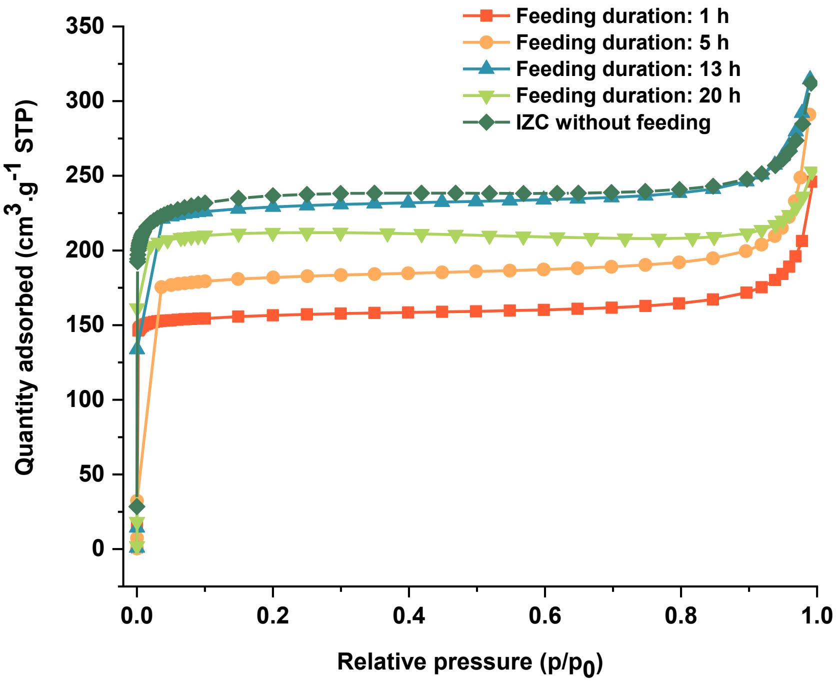

Fig. S3. N_2 adsorption isotherms of FB-made CHA zeolites with different feeding durations. The final molar composition of zeolites after feeding was 1 SiO_2: 0.012 AlO_2H: 0.17 TMAdaOH: 27.47 H_2O.

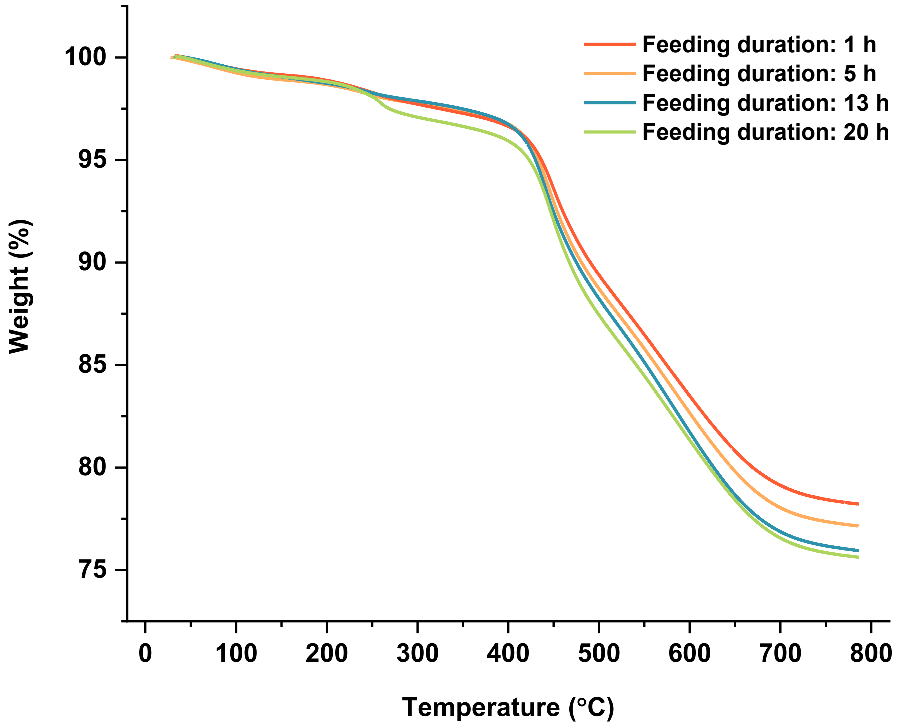

Fig. S4. TGA curves of FB-made CHA zeolites with different feeding durations. The final molar composition of zeolites after feeding was 1 SiO_2: 0.012 AlO_2H: 0.17 TMAdaOH: 27.47 H_2O.

Table S1. Micropore volume of FB-made CHA zeolites derived from N_2 physisorption analysis.

Sample name	Micropore volume of the final product ($cm^3.g^{-1}$)
Feeding duration: 1 h	0.24
Feeding duration: 5 h	0.25
Feeding duration: 13 h	0.32
Feeding duration: 20 h	0.31
IZC without feeding	0.32

Section S2. Effect of different cooling approaches on Si/Al_{ICP} ratios

Contrary to the high-silica results achieved in the main text, the Si/Al ratio of samples with timed-cooling was lower, i.e., around 70 as analyzed with ICP method (Fig. S5). Although it is still higher compared to standard IZC, but lower than the previously synthesized samples with the instant cooling approach.

Several factors may contribute to these controversial observations:

- Fast addition of aqueous Si source dilutes the system rapidly, temporarily affects the supersaturation level, and may delay some Al species from incorporating into the framework. However, using a timed cooling procedure allows Al species more opportunity to incorporate into the solid framework during the extended crystallization phase.
- The added colloidal silica may not fully dissolve into monomeric silicates but participates directly in the crystallization process, potentially acting as siliceous domains within the synthesis medium. These domains may either contribute to framework growth or remain partially unincorporated, which could promote the formation of internal silanol defects.
- From a microkinetic perspective, Si-rich droplets or local domains with temporarily high saturation could form during rapid feeding. These domains may serve as local nucleation or growth centers, similar to the behavior observed in seed-assisted synthesis, which can impact framework homogeneity and Si/Al distribution.

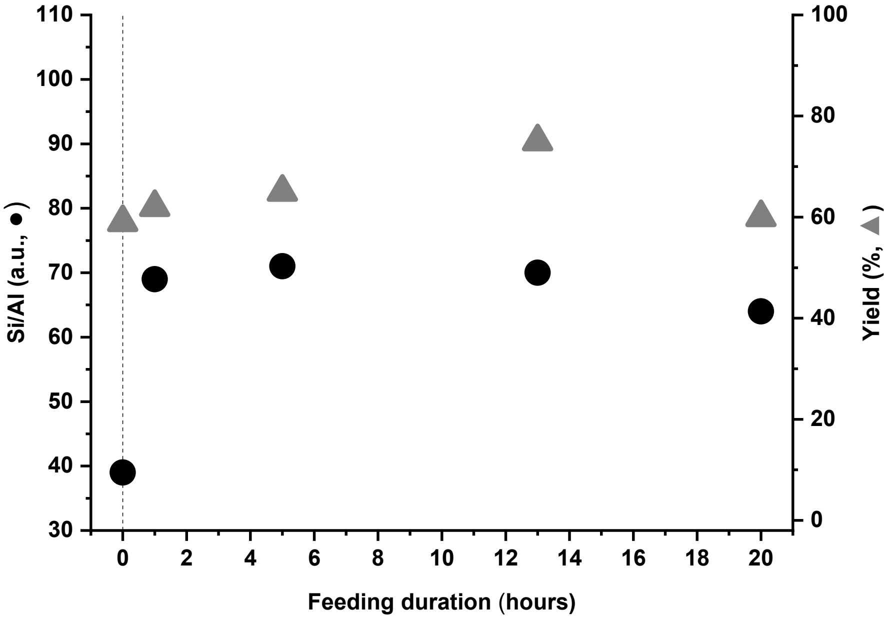

Fig. S5. Si/Al ratio and yield of FB-made CHA zeolites (timed cooling) based on different feeding durations. For this series of experiments, the reactor was cooled over a few hours (timed cooling), and then the product was collected. The final molar composition of zeolites after feeding was 1 SiO_2: 0.012 AlO_2H: 0.17 TMAdaOH: 27.47 H_2O.

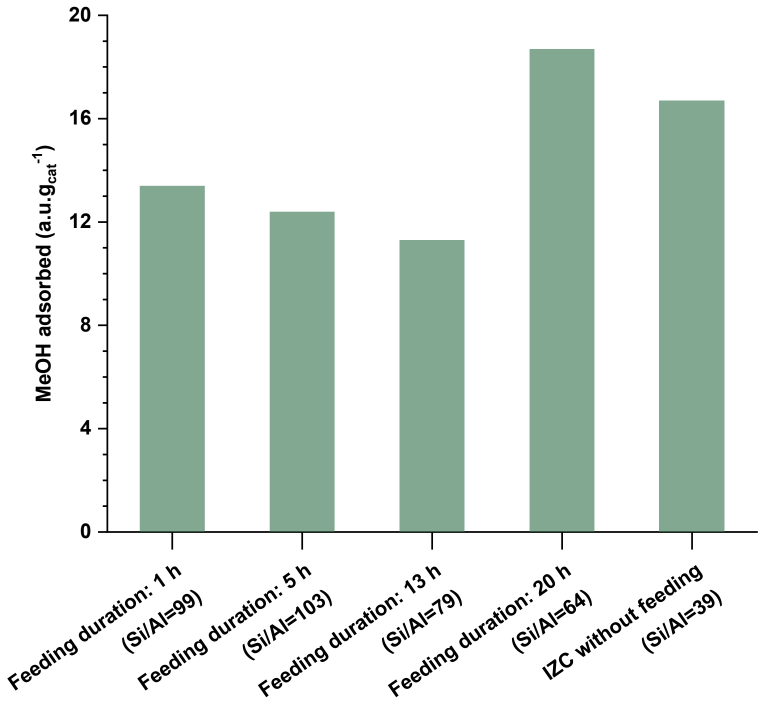

Fig. S6. The relative comparison of the MeOH adsorption capacity (extracted from MeOH FT-IR data) of FB-made CHA samples.

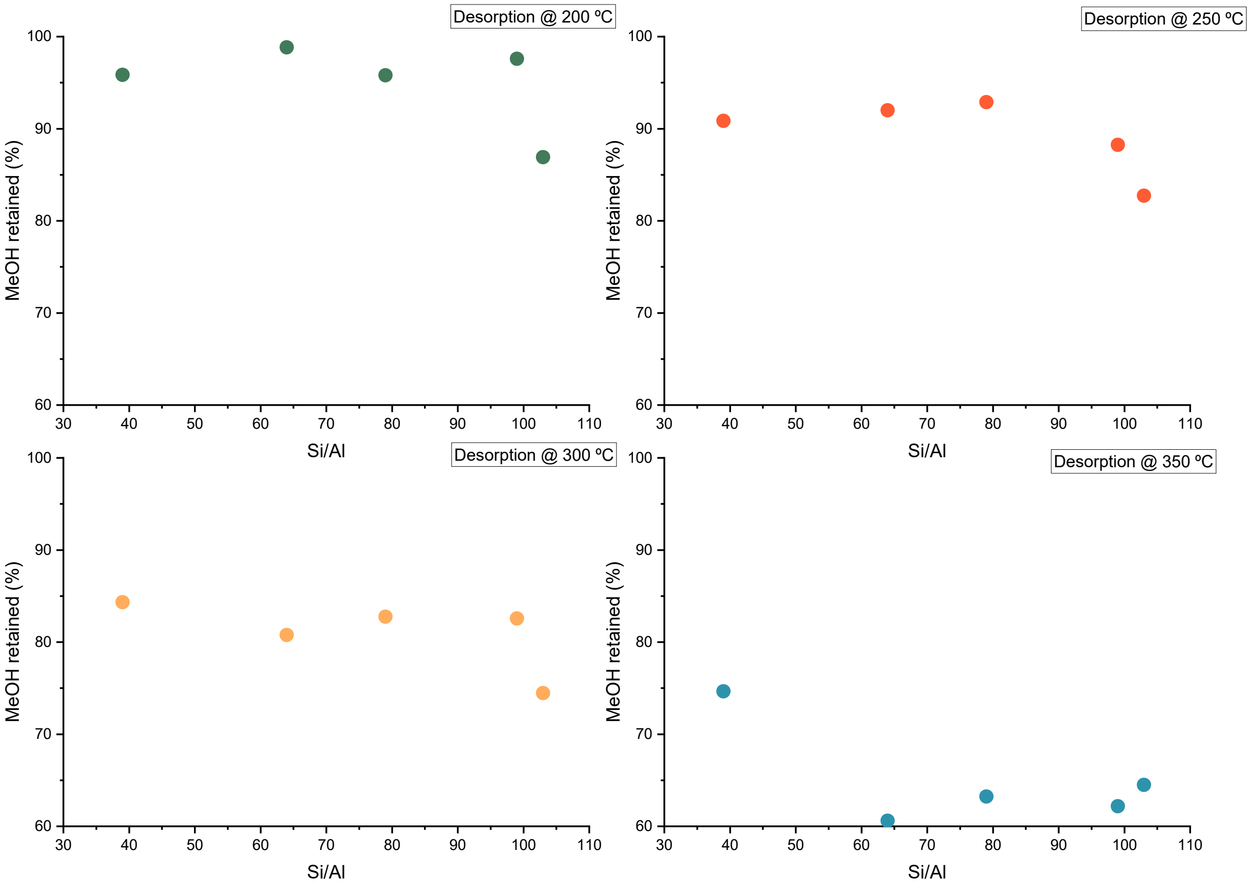

Fig. S7. Relative amount of MeOH retained (% of adsorbed amount) in FB-made CHA samples based on their Si/Al ratio at different desorption temperatures.

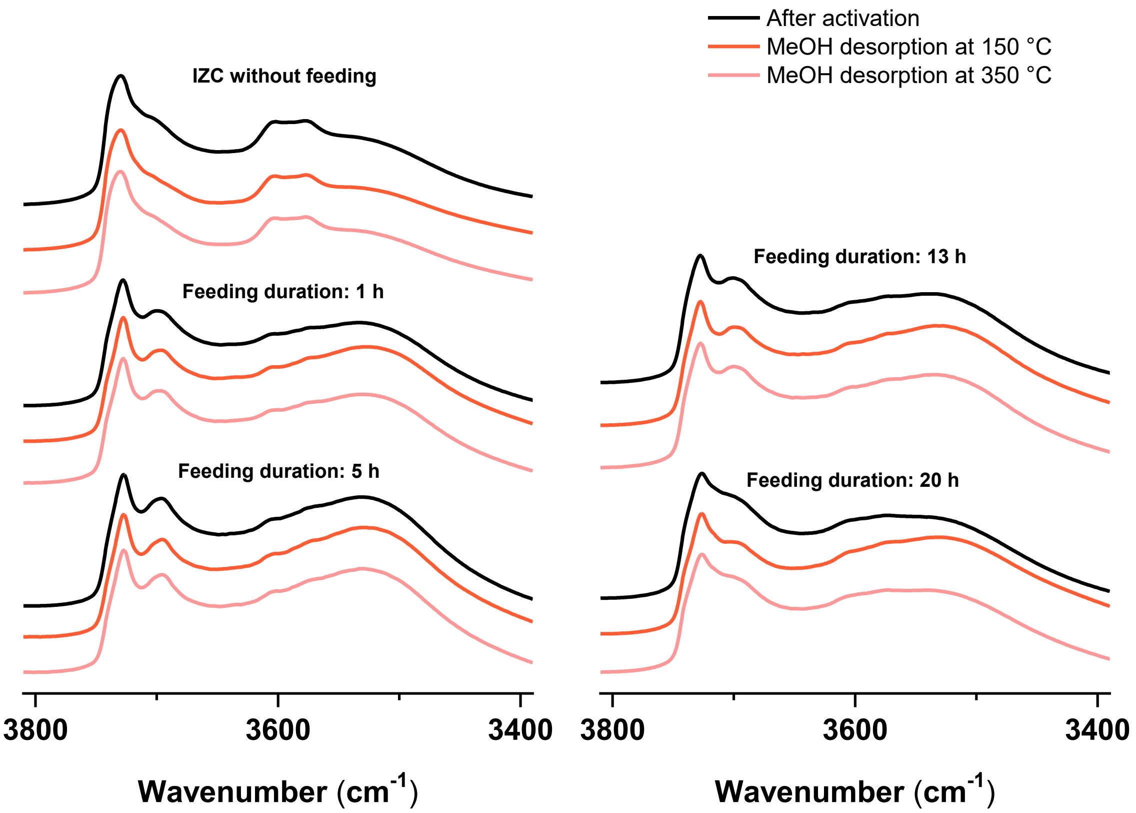

Fig. S8. FTIR spectra reporting the interaction of the FB-made CHA zeolites with MeOH at the desorption steps (150 and 350 °C) as compared to the fresh activated catalyst without any MeOH.

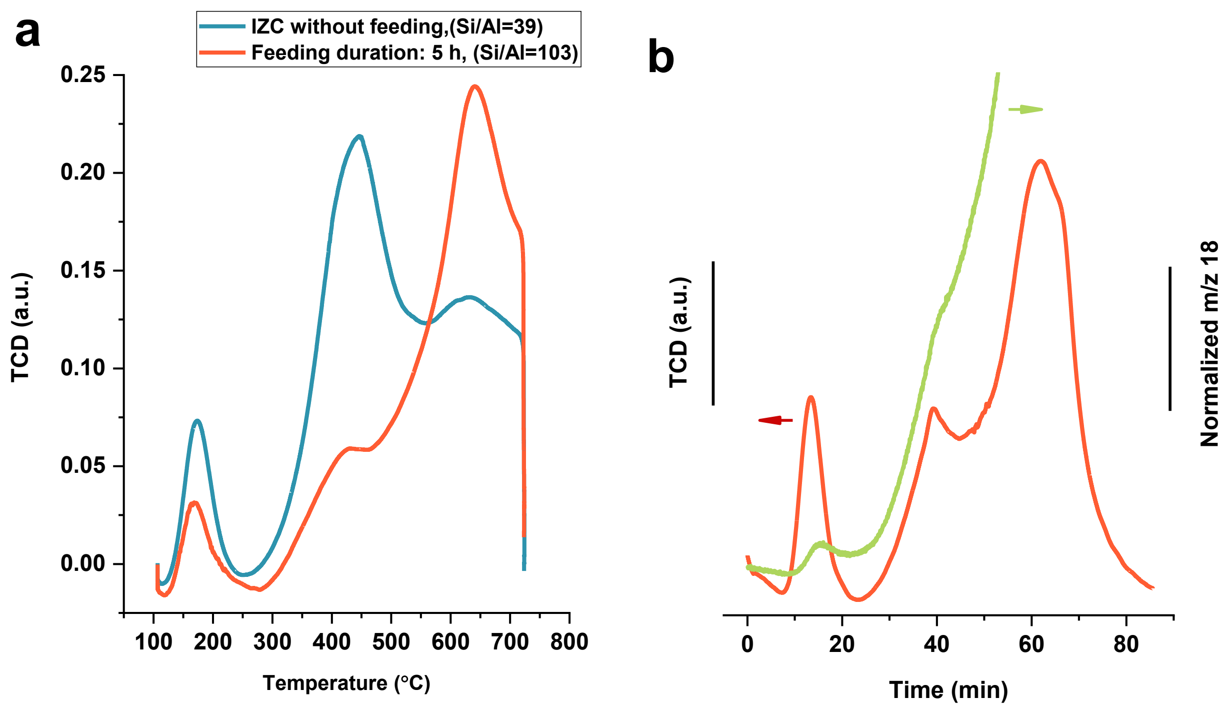

Fig. S9. (a) Comparison of TCD signals based on different temperatures for different FB samples and (b) TCD signal and MS spectra of normalized m/z 18 value over time for the FB-made sample synthesized with 5-hour feeding (Si/Al=103).

Section S3. Trials for fine-tuning the recipe of FAU-to-LEV synthesis

Table S2 presents some batch experiments to determine a suitable recipe with a relatively short synthesis time and a dilution level compatible with the FB platform. All these experiments were performed using CBV 720 (Si/Al=15) as parent zeolite and different types of OSDAs, such as Choline hydroxide, 1-Adamantanamine, or DMPOH, in 25 mL batch autoclaves with stirring.

Table S2. Trial Experiments of FAU-to-LEV in batch reactor.

**Chemical composition**	**Synthesis temperature**	**Synthesis time**	**Topology**
1 SiO_2: 0.066 AlO_2H: 0.5 Choline hydroxide: 0.2 NaCl: 5 H_2O	140 °C	5 days	Mix
		6 days	LEV+dense phase

		7 days	Mix
		8 days	Mix
1 SiO_2: 0.066 AlO_2H: 0.5 Choline hydroxide: 0.2 NaCl: 10 H_2O		6 days	FAU+dense phase
1 SiO_2: 0.066 AlO_2H: 0.5 Choline hydroxide: 0.2 NaCl: 20 H_2O		6 days	FAU+dense phase
1 SiO_2: 0.066 AlO_2H: 1 Choline hydroxide: 0.2 NaCl: 20 H_2O		6 days	FAU+LEV+dense phase
1 SiO_2: 0.066 AlO_2H: 1 Choline hydroxide: 0.2 NaCl: 30 H_2O		6 days	Amorph.
1 SiO_2: 0.066 AlO_2H: 0.3 1-Adamantanamine: 0.05 NH_4F: 10 H_2O	160 °C	4 days<br>5 days<br>6 days<br>7 days	LEV
1 SiO_2: 0.066 AlO_2H: 0.3 1-Adamantanamine: 0.05 NH_4F: 20 H_2O		4 days<br>5 days<br>6 days<br>7 days	LEV
1 SiO_2: 0.066 AlO_2H: 0.3 1-Adamantanamine: 0.05 NH_4F: 30 H_2O		4 days<br>5 days<br>6 days<br>7 days	LEV
1 SiO_2: 0.066 AlO_2H: 0.2 DMPOH: 0.3 NaOH: 10 H_2O		2 days	LEV
1 SiO_2: 0.066 AlO_2H: 0.2 DMPOH: 0.3 NaOH: 20 H_2O			Mix
1 SiO_2: 0.066 AlO_2H: 0.2 DMPOH: 0.3 NaOH: 40 H_2O			LEV+amorph.

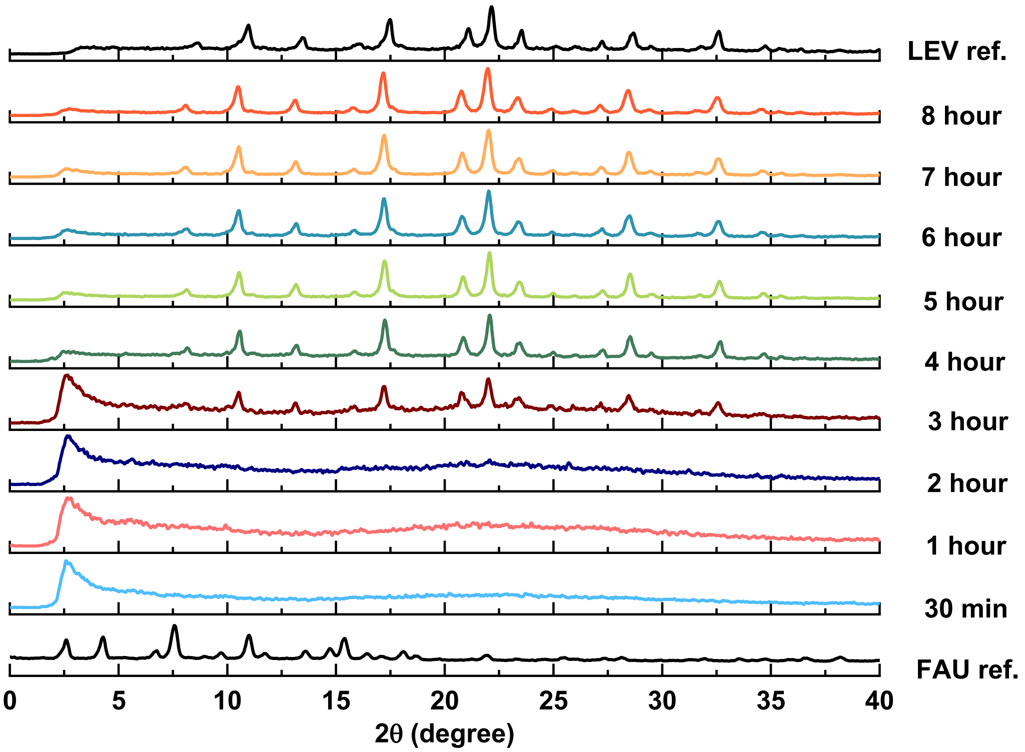

Fig. S10. XRD diffractograms of samples obtained during early stages of interzeolite conversion of FAU-to-LEV. The synthesis was performed in the FB set-up without feeding ingredients (batch mode, two runs for sampling effect minimization), and sampling was conducted to track the synthesis. The molar composition was 1 SiO_2: 0.066 AlO_2H: 0.2 DMPOH: 0.3 NaOH: 10 H_2O.

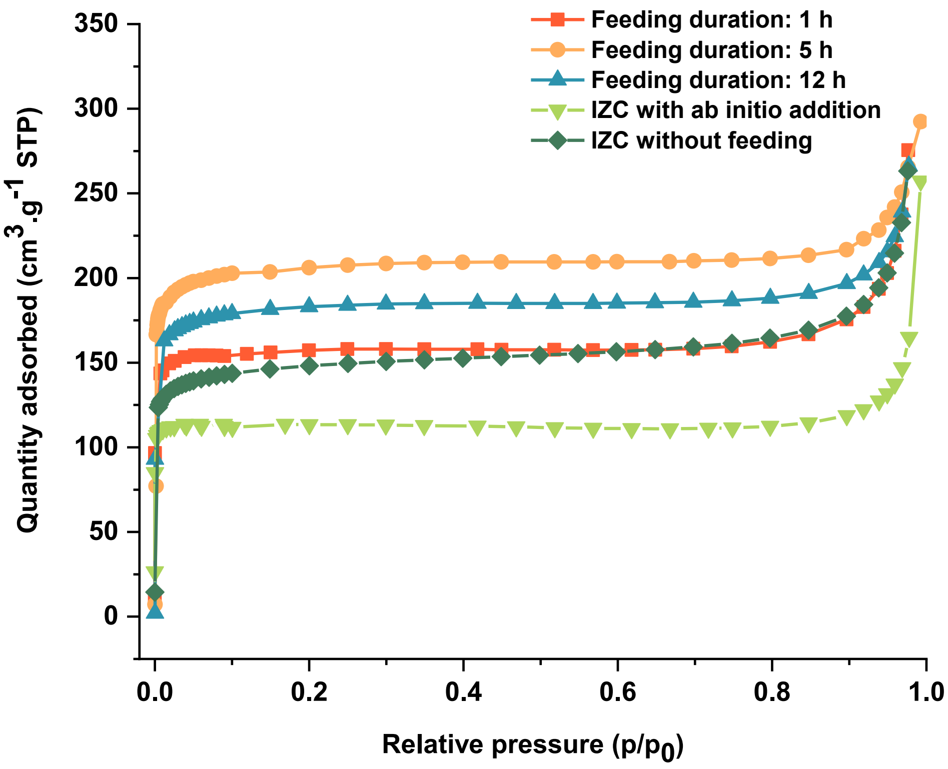

Fig. S11. N_2 adsorption isotherms of FB-made LEV zeolites. The final molar composition of zeolites after feeding was 1 SiO_2: 0.045 AlO_2H: 0.13 DMPOH: 0.2 NaOH: 16.6 H_2O.

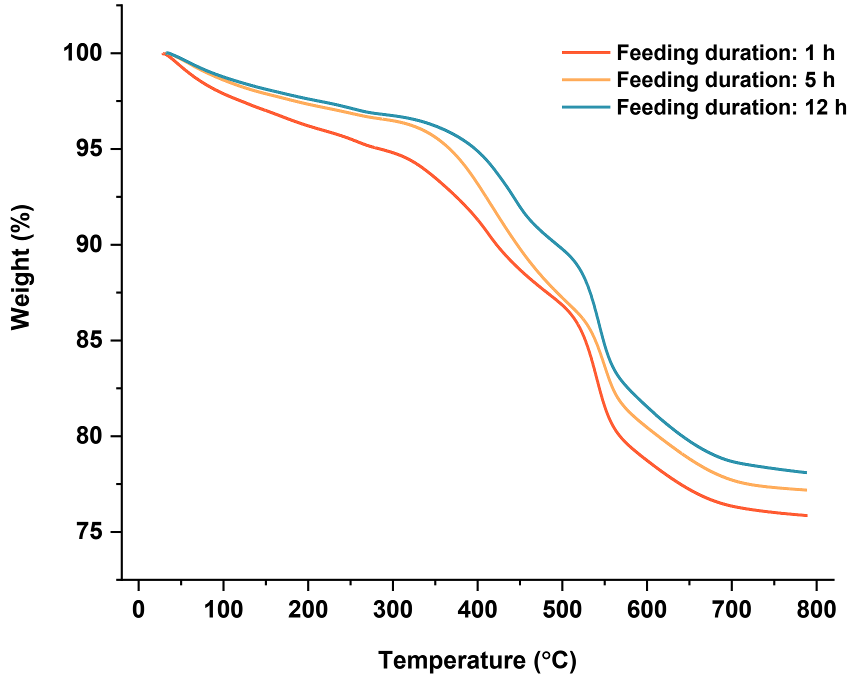

Fig. S12. TGA curves of FB-made LEV zeolites with different feeding durations. The final molar composition of zeolites after feeding was 1 SiO_2: 0.045 AlO_2H: 0.13 DMPOH: 0.2 NaOH: 16.6 H_2O.

Table S3. Micropore volume of FB-made LEV zeolites derived from N_2 physisorption analysis.

Sample name	Micropore volume of the final product ($cm^3.g^{-1}$)
Feeding duration: 1 h	0.21
Feeding duration: 5 h	0.28
Feeding duration: 12 h	0.24
IZC with ab initio addition	0.17
IZC without feeding	0.18

Section S4. Varying the amount of added Silica solution in the feeding

In LEV experiments, we used 15 mL of aqueous silica solution (10 wt%) and achieved a Si/Al ratio of 15 after 5 hours of feeding. The next standing in line question is whether we can increase the Si/Al ratio even more by either 1) using a more concentrated feeding solution or 2) adding

more volume of the current solution. The first option is not feasible on the FB platform because a more concentrated solution can cause blockages in the feeding lines. We attempted to increase the volume by following the second option and added 25 mL of silica aqueous solution instead of 15 mL in a fixed 5-hour feeding time, which allowed us to produce pure LEV with a Si/Al ratio of 20. Increasing the feeding volume to 50 mL would result in a mixed topology, rather than a pure LEV. Both XRD spectra are shown in Fig. S13. It should be noted that after calcination, there were some black spots in the zeolite with 25 mL of feeding volume, while the other sample with 50 mL of feeding volume was completely black. It suggests that a further increase in feeding volume will not guarantee an elevated Si/Al ratio in the final zeolite, and the added Si amount should be consistent with other precursors. Otherwise, it might form some thermally unstable structures or impurities.

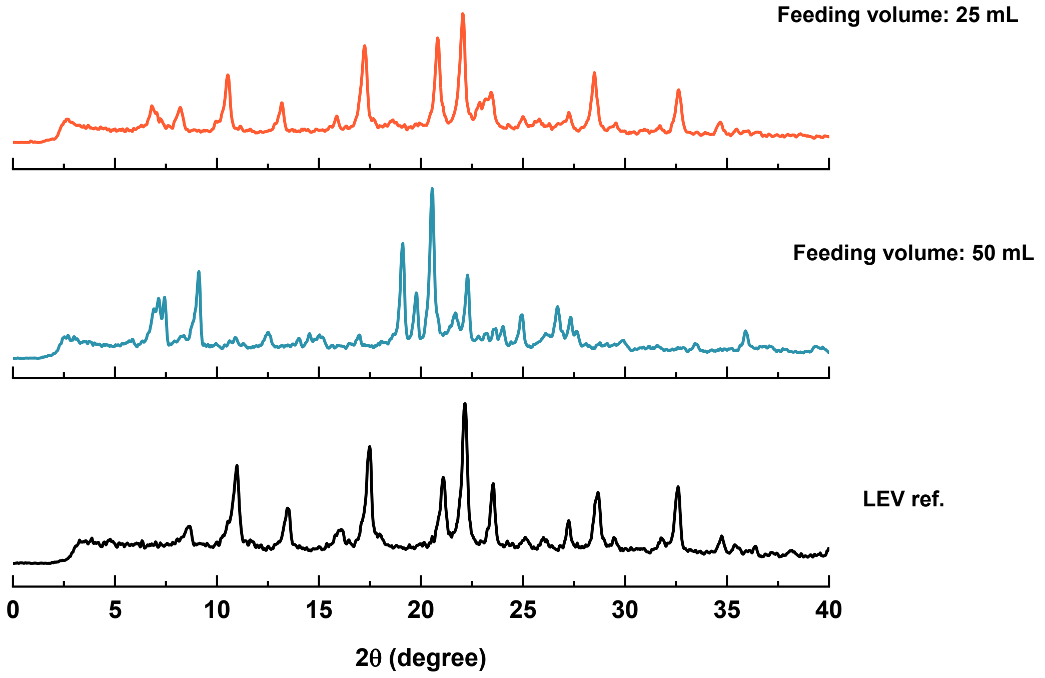

Fig. S13. XRD diffractograms of samples obtained with varying amounts of added colloidal silica solution during the interzeolite conversion of FAU to LEV at 160 °C.

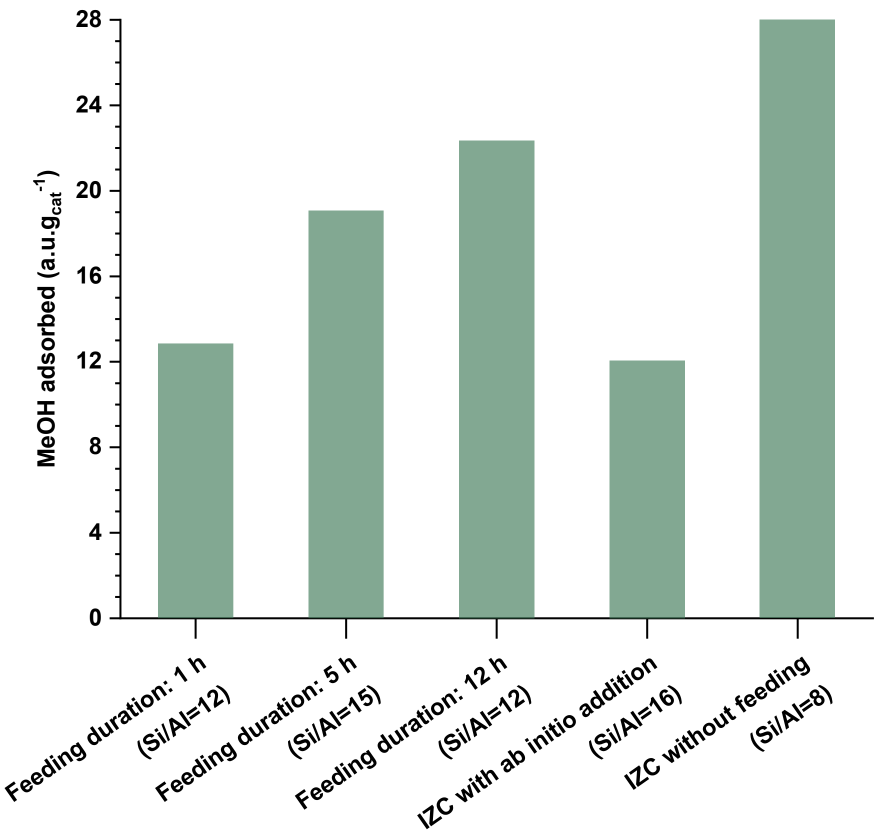

Fig. S14. The relative comparison of the MeOH adsorption capacity (extracted from MeOH FT-IR data) of FB-made LEV samples.

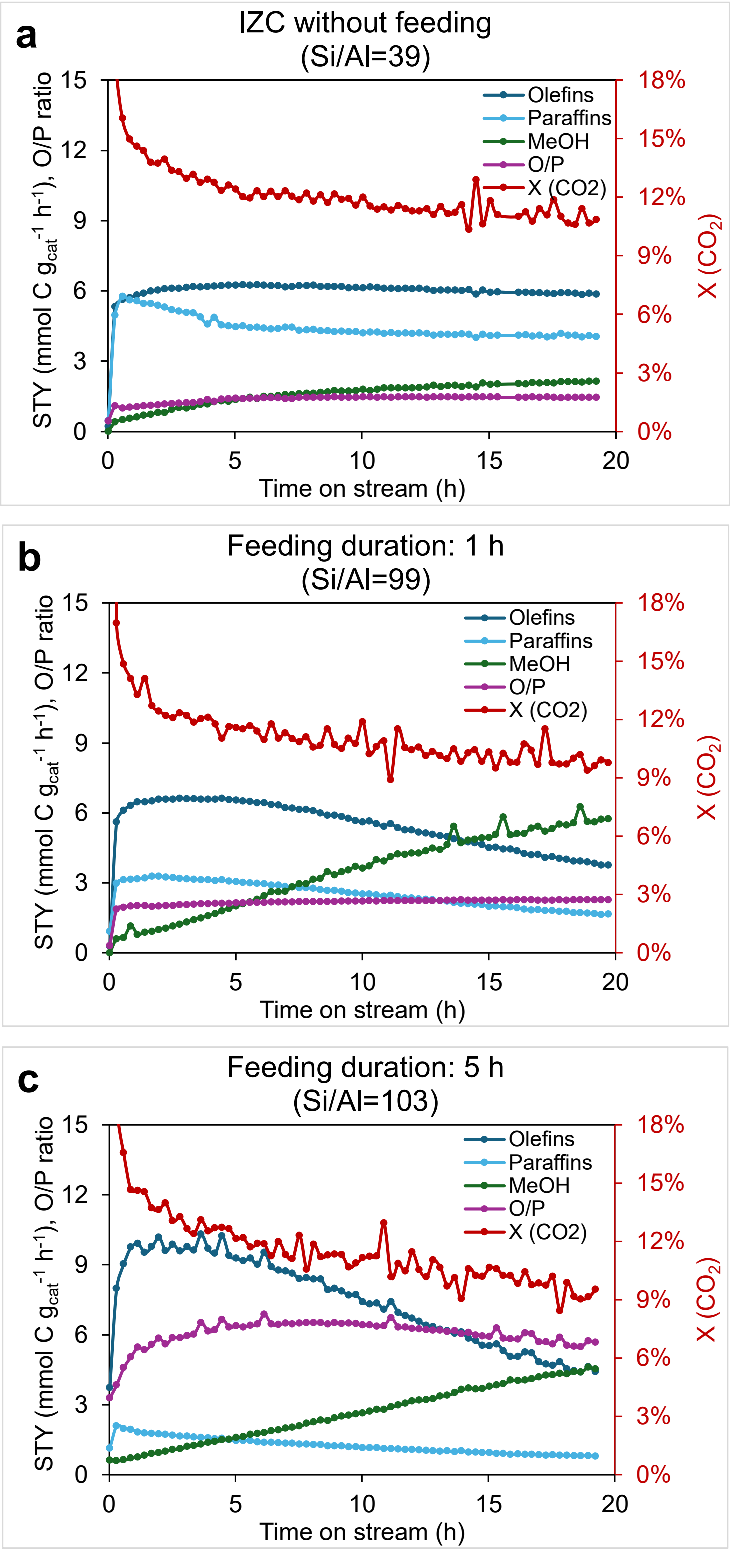
a
IZC without feeding
(Si/Al=39)
b
Feeding duration: 1 h
(Si/Al=99)
c
Feeding duration: 5 h
(Si/Al=103)
Olefins
Paraffins
MeOH
O/P
X (CO2)
STY (mmol C g$_{cat}$$^{-1}$ h^{-1}), O/P ratio
X (CO_2)
Time on stream (h)
0
3
6
9
12
15
0%
3%
6%
9%
12%
15%
18%
5
10
20

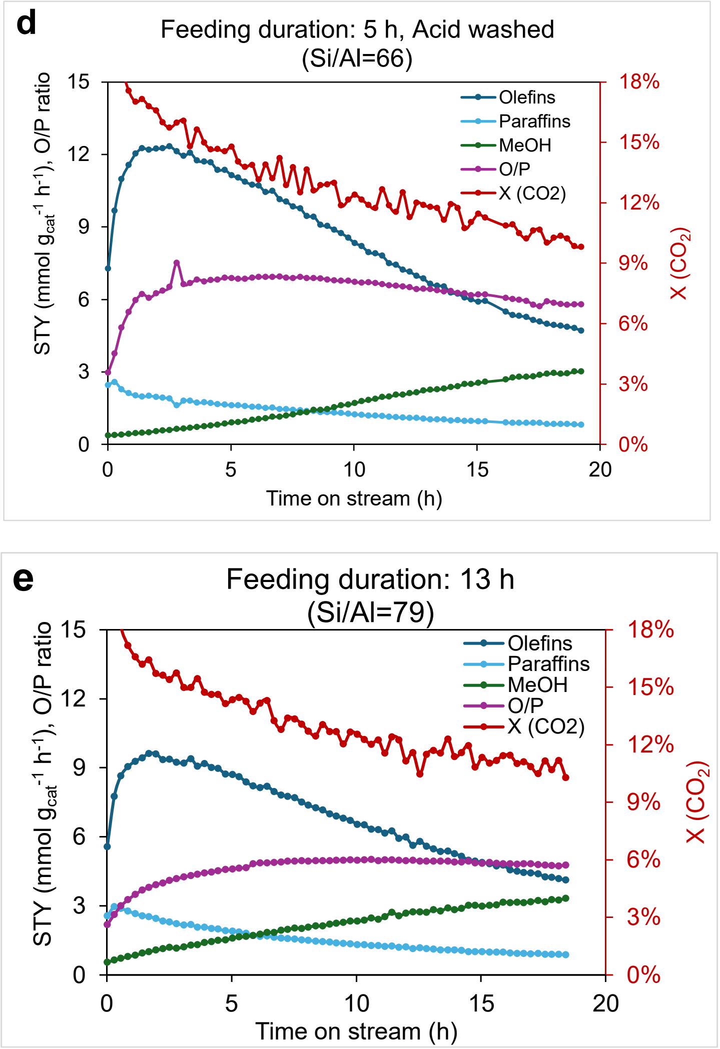

Fig. S15. CO_2 conversion, product space-time yield (STY), and olefin to paraffin ratio (O/P) as a function of time on stream (TOS) over FB-made CHA zeolites (a) without feeding, and with (b) 1 h feeding, (c) 5 h feeding, (d) 5 h feeding and acid washed, and (e) 13 h feeding. Note that DME production was not detected. Reaction conditions: T = 380 °C, P = 4.0 MPa, GHSV = 18000 ml.g_{cat}^{-1}.h^{-1}, H_2 /CO_2 = 3.0.

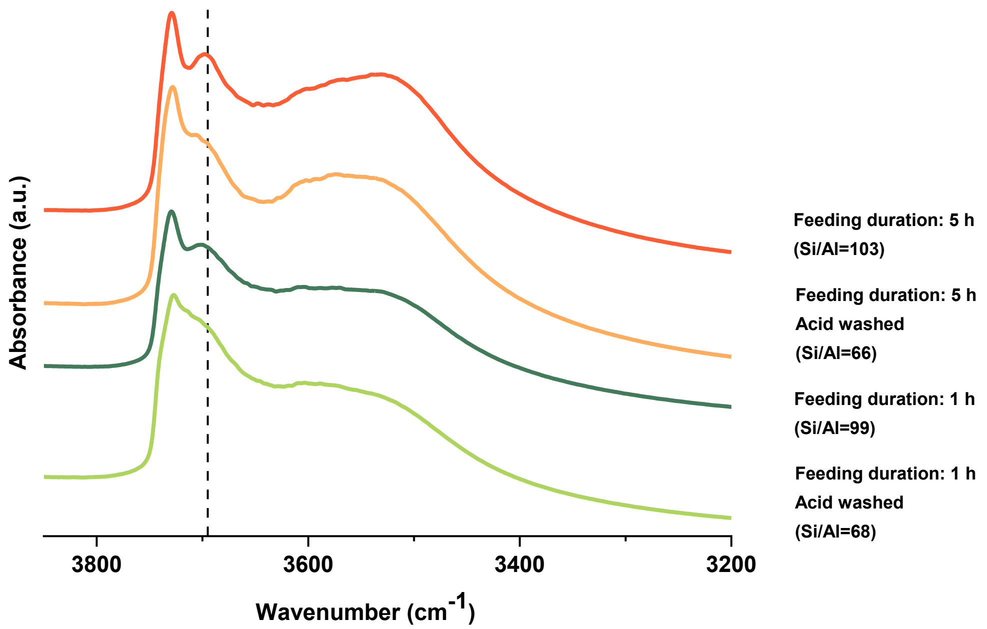

Fig. S16. FTIR spectra of FB-made CHA zeolites with different feeding durations (before/after acid washing treatment).

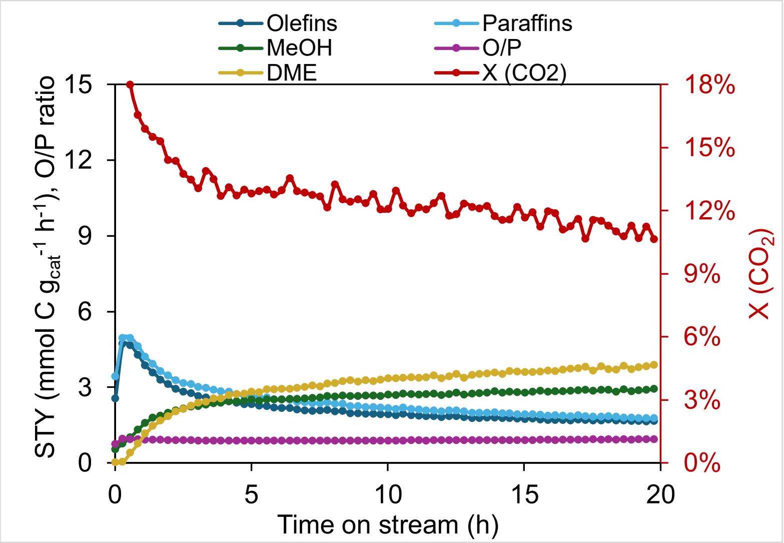

Fig. S17. CO_2 conversion, product space-time yield (STY), and olefin to paraffin ratio (O/P) as a function of time on stream (TOS) over FB-made LEV zeolite synthesized with 5 h feeding (Si/Al=16). Reaction conditions: T = 380 °C, P = 4.0 MPa, GHSV = 18000 ml.g_{cat}^{-1}.h^{-1}, H_2 /CO_2 = 3.0.

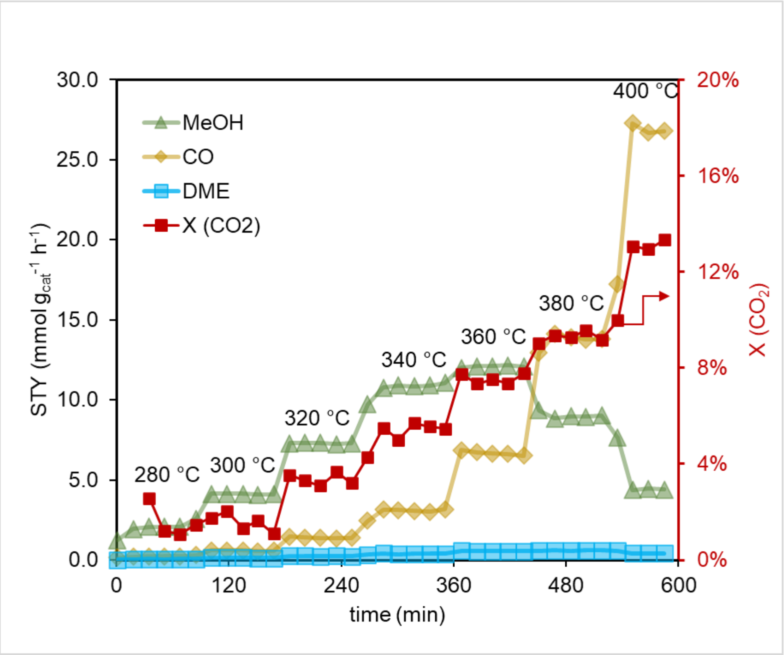

Fig. S18. CO_2 conversion and product space-time yield (STY), as a function of time on stream (TOS) over bare ZnZrOx catalyst. Reaction conditions: T = 280 - 400 °C, P = 4.0 MPa, GHSV = 27000 $ml.g_{cat}^{-1}.h^{-1}$, H_2 /CO_2 = 3.0.

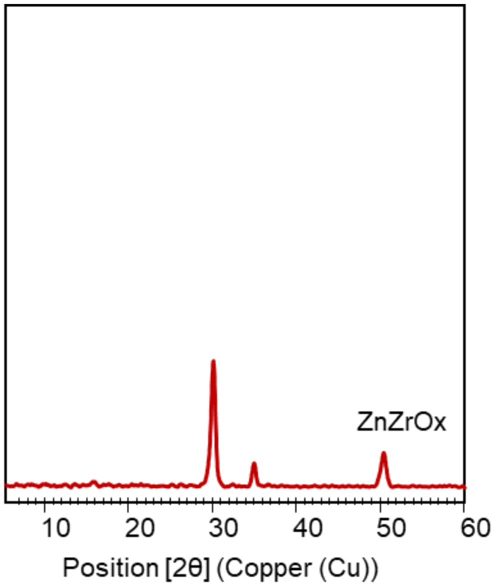

Fig. S19. PXRD diffractogram of ZnZrOx post calcination at 500 °C for 3 h.[1]

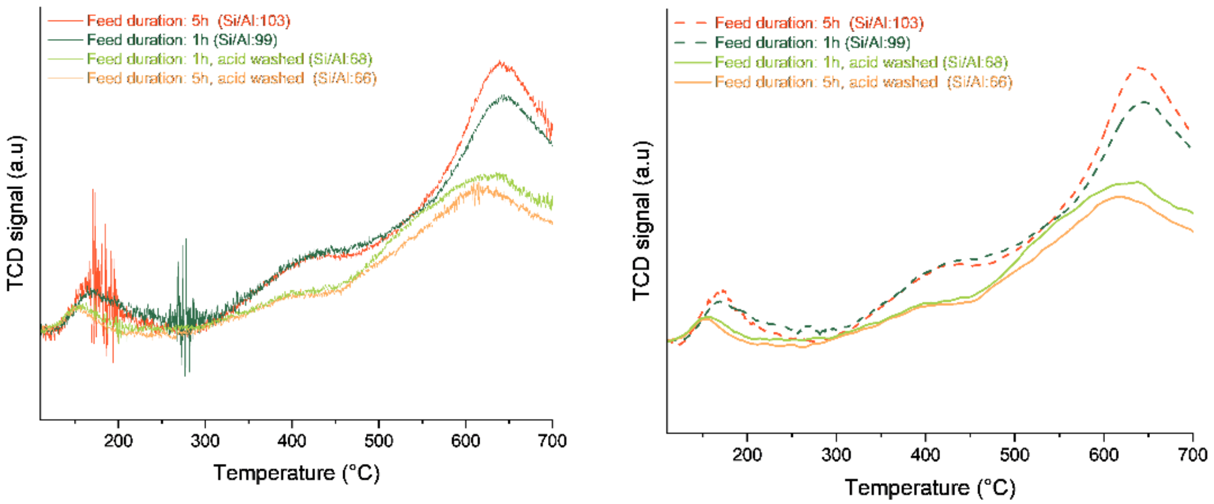

Fig. S20. NH_3-TPD profiles of samples with and without acid washing. The plot on the left shows the raw data, while the plot on the right shows the data smoothed using the Savitzky-Golay method with a 300-point window to reduce noise. The profiles highlight the reduced acidity of the acid-washed samples, as well as the lower NH3 desorption temperatures.

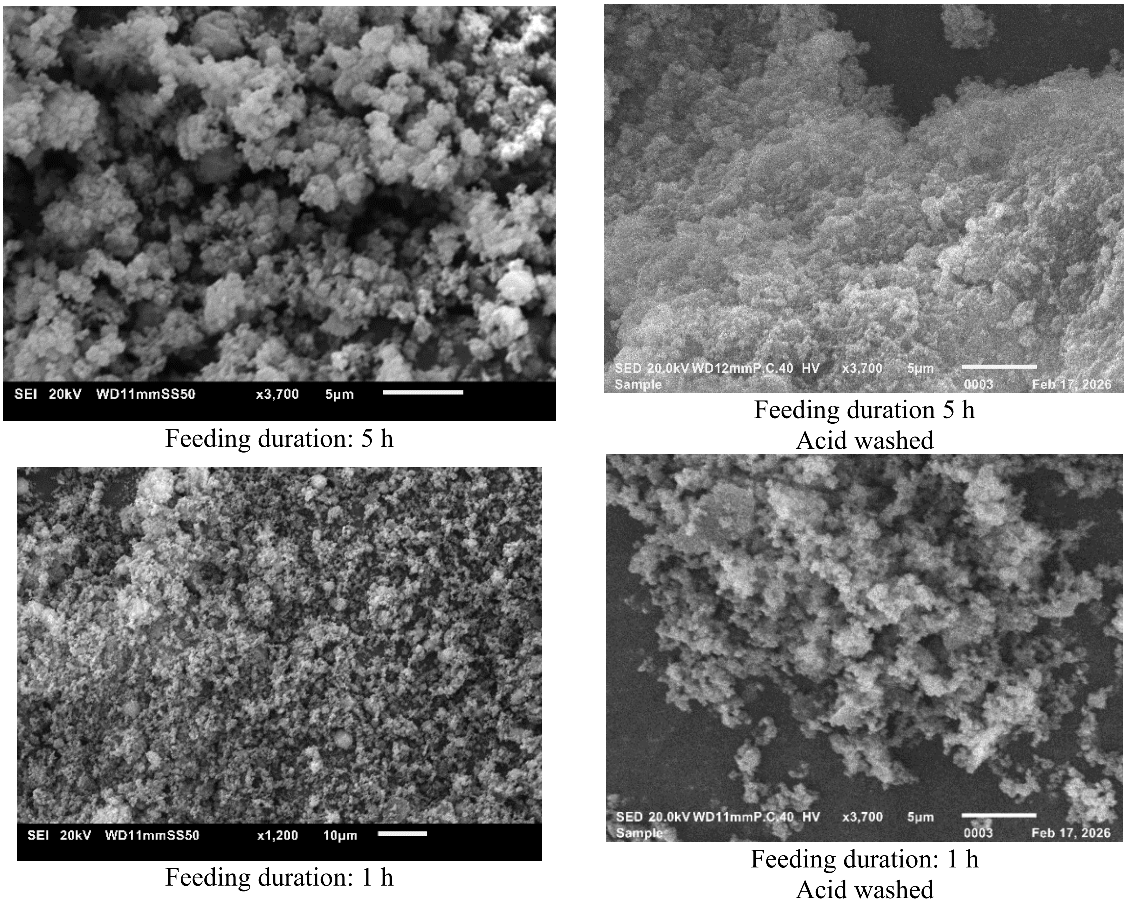

Fig. S21. SEM images of FB-made CHA zeolites with different feeding durations (before/after acid washing treatment). SEM was performed on the JEOL JSM-6010LV microscope at an acceleration voltage of 15 or 20 kV.

1 A. Sajid, J. Devos, S. Robijns, T. Donckels, I. Khalil and M. Dusselier, *J. Catal.*, 2025, **442**, 115927.
